# Behavioral Monitoring of Sexual Offenders Against Children in Virtual Risk Situations: A Feasibility Study

**DOI:** 10.3389/fpsyg.2018.00224

**Published:** 2018-03-06

**Authors:** Peter Fromberger, Sabrina Meyer, Kirsten Jordan, Jürgen L. Müller

**Affiliations:** Clinic for Psychiatry and Psychotherapy – Forensic Psychiatry, Human Medical Center Göttingen, Georg-August-University Göttingen, Göttingen, Germany

**Keywords:** risk assessment, risk management, virtual reality, virtual environment, child abuser, pedophilic disorder, pedophilia

## Abstract

The decision about unsupervised privileges for sexual offenders against children (SOC) is one of the most difficult decisions for practitioners in forensic high-security hospitals. Facing the possible consequences of the decision for the society, a valid and reliable risk management of SOCs is essential. Some risk management approaches provide frameworks for the construction of relevant future risk situations. Due to ethical reasons, it is not possible to evaluate the validity of constructed risk situations in reality. The aim of the study was to test if behavioral monitoring of SOCs in high-immersive virtual risk situations provides additional information for risk management. Six SOCs and seven non-offender controls (NOC) walked through three virtual risk situations, confronting the participant with a virtual child character. The participant had to choose between predefined answers representing approach or avoidance behavior. Frequency of chosen answers were analyzed in regards to knowledge of the participants about coping skills and coping skills focused during therapy. SOCs and NOCs behavior differed only in one risk scenario. Furthermore, SOCs showed in 89% of all cases a behavior not corresponding to their own belief about adequate behavior in comparable risk situations. In 62% of all cases, SOCs behaved not corresponding to coping skills they stated that therapists focused on during therapy. In 50% of all cases, SOCs behaved in correspondence to coping skills therapists stated that they focused on during therapy. Therapists predicted the behavior of SOCs in virtual risk situations incorrect in 25% of all cases. Thus, virtual risk scenarios provide the possibility for practitioners to monitor the behavior of SOCs and to test their decisions on unsupervised privileges without endangering the community. This may provide additional information for therapy progress. Further studies are necessary to evaluate the predictive and ecological validity of behavioral monitoring in virtual risk situations for real life situations.

## 1. Introduction

The decision about unsupervised privileges for sexual offenders against children (SOC) is one of the most difficult decisions for practitioners in forensic high-security hospitals. If a positive decision is made too early in the therapy process, the risk for the community in the form of a re-offense is too high. If it takes place too late, the patient will be incarcerated unnecessarily long and the therapy process may stop, e.g., preventing the transfer of more functional coping strategies to extramural settings. In order to mitigate possible consequences of an early release, for both the community as well as the patients, an accurate and reliable risk management is essential.

### 1.1. Risk management of sexual offenders against children: state-of-the-art

The most prominent risk management model is the Risk-Need-Responsivity (RNR)-Model (Andrews and Bonta, 2010). The starting point of every risk management following the RNR is a valid and reliable risk assessment of static risk and dynamic factors (Risk principle). The treatment should focus on stable and acute dynamic risk factors (Need principle). At last, treatment should consider the individual skills and potentials of each SOC, in order to use treatment methods convenient for the individual SOC (Responsivity principle). Thus, the risk assessment guides the therapy (Andrews and Bonta, 2010).

Static risk factors are not changeable and include demographic features (e.g., age) and historical events (e.g., past offenses). Dynamic risk factors are theoretically changeable (e.g., antisocial attitudes) and are called criminogenic needs within the RNR-Model. Dynamic risk factors can be further divided into stable and acute dynamic risk factors, differentiated by their stability over time. Stable dynamic risk factors are changing within weeks or months (e.g., cognitive distortions), whereas acute dynamic risk factors are changing within hours or days (Heffernan and Ward, 2015). The risk assessment should be based on empirically confirmed risk factors assessed with actuarial risk assessment tools (Craig and Rettenberger, 2016). Actuarial risk assessment tools exist to assess static risk factors of SOCs (e.g., Static-99, Harris et al., 2003) as well as dynamic risk factors (e.g., Stable-2007, Hanson and Harris, 2007b; Acute-2007, Hanson and Harris, 2007a). They demonstrate moderate to good prospective validity. These risk assessment tools for dynamic risk factors are categorized as the third generation of risk assessment tools (Craig and Rettenberger, 2016).

Recently, it was criticized that the concept of dynamic risk factors as criminogenic needs within the RNR-Model assumes, that dynamic risk factors are causal factors for a sexual recidivism. However, from an empirical point of view, there only seems to be a correlation, and no causal relationship between dynamic risk factors and recidivism. Consequently, it is uncertain if focusing only on dynamic risk factors is the most efficient therapeutic approach (Heffernan and Ward, 2015; Ward, 2015). Other authors conceptualize dynamic risk factors as vulnerability factors, which must be evaluated in the situational context. This means that the existence of one or more dynamic risk factors causes not necessarily a sexual child offense, but it can pave the way to an offense if specific contextual factors are simultaneously present (Mann et al., 2010). Thus, the knowledge about the existence of one or more dynamic risk factors is only meaningful, if the contextual factors of a possible situation in which the relapse could occur are also known. Furthermore, protective factors can interact with contextual factors and dynamic risk factors in such a way that the dynamic risk factors are (partly) abolished (Lehmann et al., 2016). To summarize, the mentioned third-generation risk assessment tools “offer no clear support to decision-making about the optimal nature of risk management or the conditions in which nature and level of risk may alter” (Logan, 2016, p. 85). As a result, risk assessment tools of the third generation cannot provide sufficient information about the behavior of SOCs in concrete situations (as they may happen during, e.g., a first walk outside of the controlled environment). Thus, for clinical decisions about unsupervised hospital privileges, information about the current therapy status and the possible individual risk situation, which may occur during unsupervised privileges, seem to be essential.

The structured professional judgment (SPJ) approach to clinical risk assessment and management of sexual offender is one example for a risk management approach which explicitly considers the current therapy status and potential risk situations (Hart and Logan, 2011; Logan, 2016). The SPJ approach comprises six distinct evaluation steps in order to provide a systematic, evidence-based and transparent clinical decision-making. In a first step, relevant information is gathered following the above mentioned risk assessment tools as well as information about the behavior in controlled environments and the therapy status. In a second step, those risk factors are identified, which are present and to what degree they are actually present. In a third step, the practitioners determine whether and to what extend the identified present risk factors are relevant for the occurrence of future offenses. In a fourth step, relevant identified risk factors are added to clinical judgments about potential protective factors, which are important for the individual case. Next, all the information gathered in the first four steps are then summarized and explicitly formulated in detailed future scenarios, which consider the individual vulnerability and risk factors as well as triggers and protective factors, in order to explain why the individual offender is at risk and under what circumstances he is at risk. Lastly, based on the identified risk scenarios, judgments about the treatment, supervision, monitoring and victim safety planning are made (Logan, 2016). In summary, the SPJ provides a detailed description of possible risk situations for the individual offender. With these constructed specific risk situations, the practitioner is enabled to make transparent and evidence-based decisions about the risk of an individual offender in a specific situation as it could occur during unsupervised privileges.

The SPJ approach seems to be able to provide—in comparison to the RNR approach—more helpful information for clinical decisions about unsupervised privileges. However, the SPJ approach also depends on theoretical assumptions based on clinical judgments of the practitioner. For example, the practitioner can only hypothesize that the therapy progress of the patient has reached the point, at which the patient is able to transfer the learned coping skills to situations outside of the controlled environment during unsupervised privileges. Here, the practitioner has to rely on (unstructured) clinical judgments, since—to the best of our knowledge—no structured tool exists for this exemplary case. Nevertheless, due to ethical reasons, it would not be possible to evaluate the clinical judgment before approving unsupervised privileges. Obviously, recent risk management models or risk assessment tools cannot solve this dilemma. Alternatively, a behavioral experiment would be the best method to assess the behavior of a patient in concrete situations outside of a controlled environment. However, it is ethically not feasible to confront a SOC with a real situation outside of the controlled environment without knowing the behavior of the patient within such a situation. One possible solution for this dilemma could be the confrontation of SOCs with virtual situations. For example the virtual simulation could be of risky situations that could potentially occur during unsupervised privileges.

### 1.2. Virtual reality in the context of sexual offenders against children

Virtual Reality (VR) can be defined as an “advanced form of human-computer interface, that allows the user to interact with and become immersed in a computer-generated environment [(virtual environment, VE)] in a naturalistic fashion” (Schultheis and Rizzo, 2001, p. 298). The most important aspect of VR is the possibility to induce presence. One widely accepted definition of presence describes it as the feeling of being in one place or environment even when one is physically situated in another (Schuemie et al., 2001). The concept of presence has been considered as central in VR research, because it assumes that a higher presence results in the same emotions and reactions within a VE which would be expected in a similar real-world situation (Alsina-Jurnet et al., 2011). Moreover, the sense of presence and its emotional engagement is assumed to be crucial for the potential of VR to catalyze “personal change because it offers a world where the individual can stay and live a specific experience” (Riva et al., 2016, p. 5). Personal change is one of the most important effects psychotherapy tries to produce. VR seems to provide a higher self-reflectiveness than provided by memory or imagination and can be as effective as reality in inducing emotional responses. This may be one of the reasons for the effectiveness of VR based treatments of anxiety disorders, post-traumatic stress disorders and phobias (Riva et al., 2016). Furthermore, VR is able to induce disorder-relevant emotions and can be sucessfully used to train coping skills in risk situations, e.g., in the context of addiction (Bordnick et al., 2011, 2012).

Despite the successful application of VR in a wide variety of psychiatric disorders and the obvious potentials VR provides, the use of VR in the context of criminology and forensic psychology is sparse (Ticknor and Tillinghast, 2011; Benbouriche et al., 2014). For forensic psychiatry, VR provides some advantages that transcend the above-mentioned general advantages of VR for psychiatry and psychology. Most important seems to be the unique possibility to expose offenders with and to train coping skills in virtual situations, which are able to elicit disorder-relevant behavior—without endangering others (Fromberger et al., 2014). In addition, the fact that realistic VEs are able to provide physical, social or emotional triggers that are able to influence the self-regulation of the user, seems to be an important advantage of VR with regards to forensic psychiatry: self-regulation abilities play an important role for the offender behavior. In their research Benbouriche et al. defined self-regulation as “the ability to change, inhibit or reorient automatic responses in order to achieve long-term objectives and thus to distance oneself from immediate environmental factors” (Benbouriche et al., 2014, p. 1, see also: Bauer and Baumeister, 2011). Contextual triggers are assumed to influence the self-regulation abilities of the offender and can result in a decrease of self-regulation abilities, which itself can cause an offense. By providing highly salient trigger, VR allows the evaluation of offenders self-regulation abilities (Benbouriche et al., 2014).

Up to now, only a few studies used VR in the context of forensic psychiatry research. Most of the studies concentrated on the usability of VR for the assessment of deviant sexual interests, mainly of SOC (Fromberger et al., 2015). For example, Renaud et al. (2014) demonstrated that high-immersive visual stimuli surpassed auditive stimuli regarding their effectiveness at inducing sexual arousal assessed with Penis-Plethysmography (PPG). 22 SOCs and 42 healthy males took part in the study. While both stimulus modalities generated significantly different genital arousal profiles for SOCs and healthy males, the VR modality provided significantly higher classification accuracy. The authors concluded that in comparison to audio stimuli, the VR system makes it possible to improve not only accuracy of group classification but also discriminant validity. In another study, Renaud et al. (2012) presented 13 male SOCs and 29 male NOCs with animated virtual characters (male and female adult, male and female child, neutral character with no texture) in a high-immersive VE for 90 seconds. SOCs showed significantly higher penile responses when facing child characters in comparison to the control group. An analysis of eye movements showed that child molesters looked significantly longer at sexual features of all virtual characters than control participants did. Thus, immersive VR in combination with psychophysiological measures seems to be a powerful tool for the assessment of deviant sexual interests, especially due to the high ecological validity of virtual characters. Recently, Fromberger et al. (2015) compared in a direct manner high-immersive VR with a conventional standard desktop system regarding their capability to measure sexual interests. The authors used the viewing time method, a well-known approach to assess (deviant) sexual interest in an indirect manner. The viewing time method uses pictures of adults and children, which are presented on a computer screen. The participants have to rate the pictures with regards to their sexual attractiveness. Without the knowledge of the participant, the time from stimulus onset until the end of the rating is assessed. This time is called Viewing Time (VT). Several studies demonstrated good classification accuracy between NOCs and SOCs as well as between homosexual and heterosexual participants (Schmidt et al., 2017). In the study of Fromberger et al. (2015) 20 homosexual and 25 heterosexual men took part. In three experimental conditions, which differed in their ability to induce presence, virtual characters of male and female were presented. The participants had to rate the virtual character with regards to their sexual attractiveness. Without the knowledge of the participant, the VT was measured. Therefore, in each experimental condition the VT paradigm to measure sexual interest was applied. Results showed, that high-immersive VT can enhance the sexual salience of virtual characters as well as the classification accuracy of the VT paradigm (Fromberger et al., 2015).

In summary, all the above-mentioned studies showed, that VR could provide more salient sexual triggers than conventional computer systems or 2D-pictures or sounds. Bearing that in mind, VR seems to be a promising tool to evaluate self-regulation abilities of (sex) offenders as proposed by Benbouriche et al. (2014).

### 1.3. Development of virtual situations for the risk management of sexual offenders against children

The development of meaningful virtual situations for SOCs in the context of risk management requires knowledge about dynamic risk factors as well as about current therapeutic approaches. As mentioned above, risk management models for SOCs do not provide sufficient information about the therapy progress and about the ability of the SOC to transfer learned coping strategies into real life situations. Thus, virtual situations require the ability of the participants to perform adequate coping strategies. Currently, treatment programs based on the RNR model seem to be the best evaluated and most successful therapeutic approach for SOCs (Hanson et al., 2009). However, no evidence-based treatment program does exist for sex offenders and studies evaluating the outcome of treatment programs are sparse and often suffer methodological problems (Carter and Mann, 2016).

One of the most influential treatment approaches of the last years was the Relapse Prevention (RP) approach. The traditional RP approach can be described as “a multi-modal, cognitive-behavioral approach. Emphasis is on helping abusers learn self-management skills to prevent relapse […].” (McGrath et al., 2010, p. 38). The RP approach focuses on the individual's offense pattern, risk factors and skills needed for avoiding relapse. Thus, patients are taught to (1) be able to recognize high-risk situations with regards to a re-offense, (2) be able to avoid high risk-situations and (3) be able to develop and use skills to avoid high risk situations and/or to cope with unavoidable high-risk situations (Laws et al., 2000). Some authors criticized the RP approach in the last years. For example, it is assumed that it focuses too much on avoidance goals positive linked approach goals (Carter and Mann, 2016). More recent treatment approaches try to overcome some of the critical points of the RP approach, e.g., the Good Lives Model (GLM) or the Self-Regulation Model (SRM) (Yates et al., 2010). These treatment models also integrate the goal of teaching the offender coping skills in their treatment programs. But coping skills are now embedded in a much wider framework, considering not only skills enabling the offender to avoid re-offenses, but also skills to achieve positive goals which do not accord with a re-offense (Yates et al., 2010). Most treatment programs at least in the US and in Canada use the RP approach, despite the mentioned criticism (McGrath et al., 2010).

In summary, most sex-offender treatment programs provide techniques to teach coping skills for high-risk situations and therefore can provide a theoretical basis for the development of meaningful virtual situations for the risk management of SOCs. It seems obvious, that some coping skills are essential before permitting SOCs to leave the controlled environment. For example, the skill to cope with high-risk situations, such as access to a potential victim seems to be essential for SOCs before leaving the controlled environment. These skills provide the ability for the child offender to stop the offense progression before an offense occurs. Therefore, meaningful virtual risk situations should require the ability of the user to use coping skills. Furthermore, they should provide some trigger (e.g., access to a potential victim), in order to transform everyday-life situations to high-risk situations for SOCs. By doing so, VR based risk assessment provides for the first time the possibility to observe the behavior of SOCs in highly ecologic valid social situations: In contrast to the above mentioned risk assessment tools and intramural settings, VR allows the consideration of situational context factors, for example specific and for the individual SOC highly relevant cues and triggers, which can influence dynamic risk factors.

### 1.4. Aims and hypotheses

In the current feasibility study, we tested for the first time the possibility to use VR for the behavioral monitoring of SOCs in risk situations. The main aim of the developed VR tool was the assessment of the possible risk of SOCs to show inadequate behavior during unsupervised privileges approved for the first time. Thus, it should be possible to assess in a direct manner to which degree SOCs are able to perform coping strategies learned during therapy. It is important to note, that the method used in this study is not sufficient to assess the overall recidivism. At the psychiatric clinic, where the forensic inpatients were recruited for the current study, one of the unsupervised privileges outside the secured ward consists of a walk outside of the controlled environment for shopping in a supermarket near the forensic hospital. Usually this walk is restricted to 1 hour. Thus, we developed risk situations that can occur while shopping in a supermarket. Furthermore, the treatment rationale of the psychiatric clinic follows the RP approach. Thus, all risk situations are constructed so that coping skills are required (ability to avoid risk situations, ability to cope with unavoidable risk situations). We hypothesize, that the monitoring of SOC's behavior in virtual risk situations can provide additional information, which are not accessible by risk assessment tools based on files or questionnaires or by observation of the inpatient's behavior during therapy. In more detail, we hypothesize that virtual risk situations allow the monitoring of the ability of SOCs to use coping skills. Furthermore, from a clinical perspective, one would assume, that SOCs, who have understood the basic rationale of the RP approach during therapy, would show avoidance behavior more frequently than healthy controls (non-offender controls, NOC) (Laws et al., 2000). The second aim of the study was to test, if forensic inpatients accept the usage of VR and if the designed VEs are able to induce a high degree of presence and co-presence in forensic inpatients. We hypothesize, that forensic inpatients show the same amount of presence and co-presence during the exposure to virtual risk situations as NOCs.

## 2. Materials and methods

### 2.1. Participants

A total of seven NOCs and six SOCs took part in the study (see Table 1). NOCs were recruited by a notice posted on the campus, by social media groups and by posts in different online forums. All NOCs were without history of neurological or psychiatric illness according to DSM-5 (American Psychiatric Association., 2013).

**Table 1:** Sample overview.

	**NOCs (*n* = 7)**	**SOCs (*n* = 6)**	**Test statistic**
**Age (years)**	*M* = 26.00 (*SD* = 4.36, *range*: 21–31)	*M* = 47.67 (*SD* = 13.47, *range*: 25–67)	*t*(5.90) = −3.77, *p* = 0.010, *d* = 2.16
**Education**			
No graduation	0% (0)	17% (1)	*Fisher's Exact Test: p* = 0.002, *V* = 1.00
Middle school	0% (0)	83% (5)	
High school	57% (4)	0% (0)	
University	43% (3)	0% (0)	
**Sexual Orientation**^a^			
Heterosexual	86% (6)	67% (4)	*Fisher's Exact Test: p* = 0.706, *V* = 0.320
Bisexual	0% (0)	17% (1)	
homosexual	14% (1)	17% (1)	
**PC-Games (frequency)**			
Daily	14% (1)	17% (1)	*Fisher's Exact Test: p* = 0.086, *V* = 0.783
Once per week	43% (3)	17% (1)	
Once per month	14% (1)	0% (0)	
Once per year	29% (2)	0% (0)	
Never	0% (0)	67% (4)	
**Previous VR Experience**			
Yes	14% (1)	0% (0)	*Fisher's Exact Test: p* = 1.00, *V* = 0.268
No	86% (6)	100% (6)	

a *Sexual orientation was assessed by the Kinsey scale asking for physical contacts (Kinsey et al., 1948). The Kinsey scale represents a Likert scale ranging from zero (exclusively gynephilic) to six (exclusively androphilic)*.

All SOCs were recruited at a forensic-psychiatric hospital. Inclusion criteria for SOCs were at least one sexual assault against children (documented in criminal records) and no unsupervised hospital privileges outside the secured ward. Exclusion criteria for SOCs were an acute psychotic episode, substance abuse during the previous month, or incapability or refusal to sign informed consent. All SOCs fulfilled the diagnostic criteria for a pedophilic disorder according to DSM-5 (American Psychiatric Association., 2013). They were on average hospitalized for 6.89 years (*SD* = 6.42, range: 1.17–19.25 years) and the number of child victims was on average 11.83 (*SD* = 7.68, range: 1.0–19.0). Child victims were on average 8.21 years old (*SD* = 1.01, range: 6.7–9.5 years).

The current recidivism risk of the SOCs was assessed with two actuarial risk assessment tools, the Static-99 and the Stable-2007. The Static-99 (Harris et al., 2003; Rettenberger and Eher, 2006) is a file-based tool for the assessment of static risk factors associated with sex offender recidivism. It consists of 10 items, which define risk factors that are not changeable over time (static risk factors). The total score ranges from zero to 12, with higher scores indicating a greater risk of sexual recidivism. The Static-99 provides good inter-rater reliability and validity (Hanson and Morton-Bourgon, 2009). The SOCs demonstrated on the Static-99 on average a score of 4.83, which identified the group at the mean at a moderate recidivism risk based on static risk factors, but also at very in-homogeneous risk levels (*SD* = 2.86, range: 1–9). The Stable-2007 (Hanson and Harris, 2007b; Matthes and Rettenberger, 2008) is an interview- and file-based tool for the assessment of dynamic risk factors for sex offender recidivism. It consists of 13 items or dynamic risk factors, between zero and two. The total score ranges from zero to 26, with higher scores indicating a greater risk of sexual recidivism. Cut-off values define three qualitative risk categories (zero-3: low risk, 4–11: moderate risk, 12+: high risk). The Stable-2007 provides a good reliability and validity (Hanson and Morton-Bourgon, 2009). On average, the SOC group showed a score of 12.50 (*SD* = 3.15, range: 8–15) on the Stable-2007, which identified the group at the mean at a high recidivism risk with regard to dynamic risk factors.

All participants provided written informed consent before participating in the study. The ethics committee of the Human Medical Center Göttingen approved the study.

### 2.2. Materials

#### 2.2.1. Apparatus

The VR system comprised a head-mounted-display (HMD; Oculus Rift Development Kit 2, Oculus VR, Inc., Irvine, CA) and a motion capturing system (MoCap; PPT-X, WorldViz LLC., Santa Barbara, CA). The HMD provides stereoscopic viewing by presenting a separate picture to each eye of the participant with a refresh-rate of at least 75 Hz, resulting in an effective resolution of 960 x 1080 pixel per eye. Head-Movements were assessed by an integrated 3-Degree-of-Freedom (DOF) movement sensor combined with two MoCap-Sensors (PPT Eyes, WorldViz LLC., Santa Barbara, CA) in order to assess the position of the participant. Interaction with the VE was done with the PPT Wand (WorldViz LLC., Santa Barbara, CA), which provides access to the position data via PPT-X and several input buttons. The MoCap system comprised an eight camera setup with an effective tracking space of 4.5 × 3.0 meters. All experiments were scripted with WorldViz Vizard Toolkit® (Version 5; WorldViz LLC., Santa Barbara, CA), a python-based VR software.

#### 2.2.2. Virtual characters

Overall, five virtual adult female characters, five virtual adult male characters, five virtual child female characters and five virtual child male characters were used in the study. All virtual characters were computer-generated realistic, fully rigged three-dimensional (3D) models of clothed human beings (see Supplementary S3). Adult characters were chosen from the Complete Characters HD Set (Rocketbox Studios GmbH, Hannover, Germany). The geometry of all characters are optimized for VR applications and textured with a resolution of 2,048 × 2,048 pixel. The textures incorporate different clothing styles and skins. Child characters were modeled with the software tool MakeHuman® (Version 1.0; www.makehuman.org), rigged, textured and animated with 3ds Max® (Version 2014; Autodesk, Inc.,). Textures (2,048 × 2,048 pixel) were designed with the 2D graphical software tool GIMP® (Version 2.8; www.gimp.org). Voices of the characters were recorded by institutional members (one female and one male) and pre-processed with the software tool Audacity® (Version 2.0; www.audacityteam.org). Especially the voices of the children were pre-processed in order to achieve a believable pitch.

#### 2.2.3. Virtual environment

Two different VEs were modeled and designed: one simpler environment for the initial rating of the virtual characters (see Supplementary S1 and Supplementary S2 Initial Rating) and a virtual supermarket for the risk scenarios. All 3D models were modeled, textured, animated and optimized for VR applications with 3ds Max® (Version 2014; Autodesk, Inc.). Textures were designed with the 2D graphical software tool GIMP® (Version 2.8; www.gimp.org).

**Figure 1:**
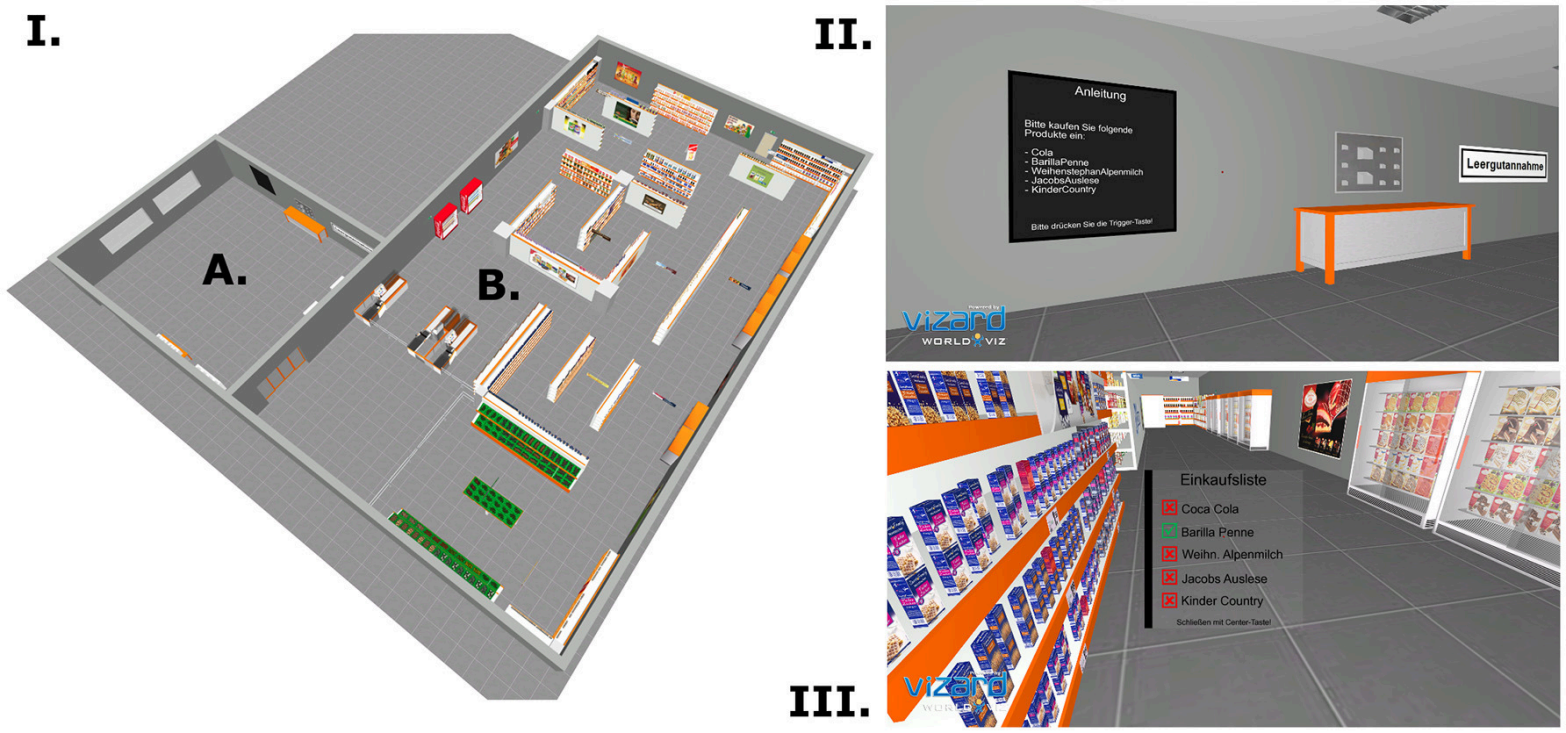
The virtual supermarket. **(I)** Bird's eye view of the virtual supermarket. The supermarket comprised an entrance area (A) and a product area (B). **(II)** Entrance area of the supermarket with the info screen. **(III)** Product area of the supermarket with the shopping list Head-up display. Red crosses symbolize products not yet in the basket; green check marks symbolize products that are already in the basket.

The virtual supermarket had a virtual size of 25.0 × 25.0 meters and represents a typical German supermarket regarding the interior, products, sounds, and structure (see Figure 1). The supermarket was comprised of two sections: the entrance area and the product area. Both areas were divided by an automatic sliding door. The entrance area consisted of some simple interior (e.g., a reverse vending machine, waste container) and a wall mounted display, in order to present feedback and instructions to the participant. The product area consisted of 30 shelves, nine fridges, three cash-points and a vegetable section. The doors of the fridges could be opened and closed by the participant. Only after opening the doors, the products in the fridges were available. Overall, more than 1,000 products were modeled and implemented in the supermarket. Interactions with the products or other objects were possible with the help of a red fixation point, the position of which was always in the middle of the screen. If the fixation point enters an interactive element, this element flashed up red and the participant could interact with the element with a specific button of the PPT Wand. Furthermore, a shopping list was shown by a Head-up display (HUD), which allowed the participant to control which products he already put into his virtual basket. The shopping list HUD could be switched on and off with a button. All interactions with virtual characters were implemented by speech and an additional HUD, which allowed the participant to choose one of the possible predefined answers. The participant had two possibilities to walk through the supermarket: Distances, which exceed the area of the lab, where handled by the joystick of the PPT Wand. Distances within the lab area could be reached by foot. Thus, larger distances were reached with the joystick, small distances per pedes.

### 2.3. Procedures

**Figure 2:**
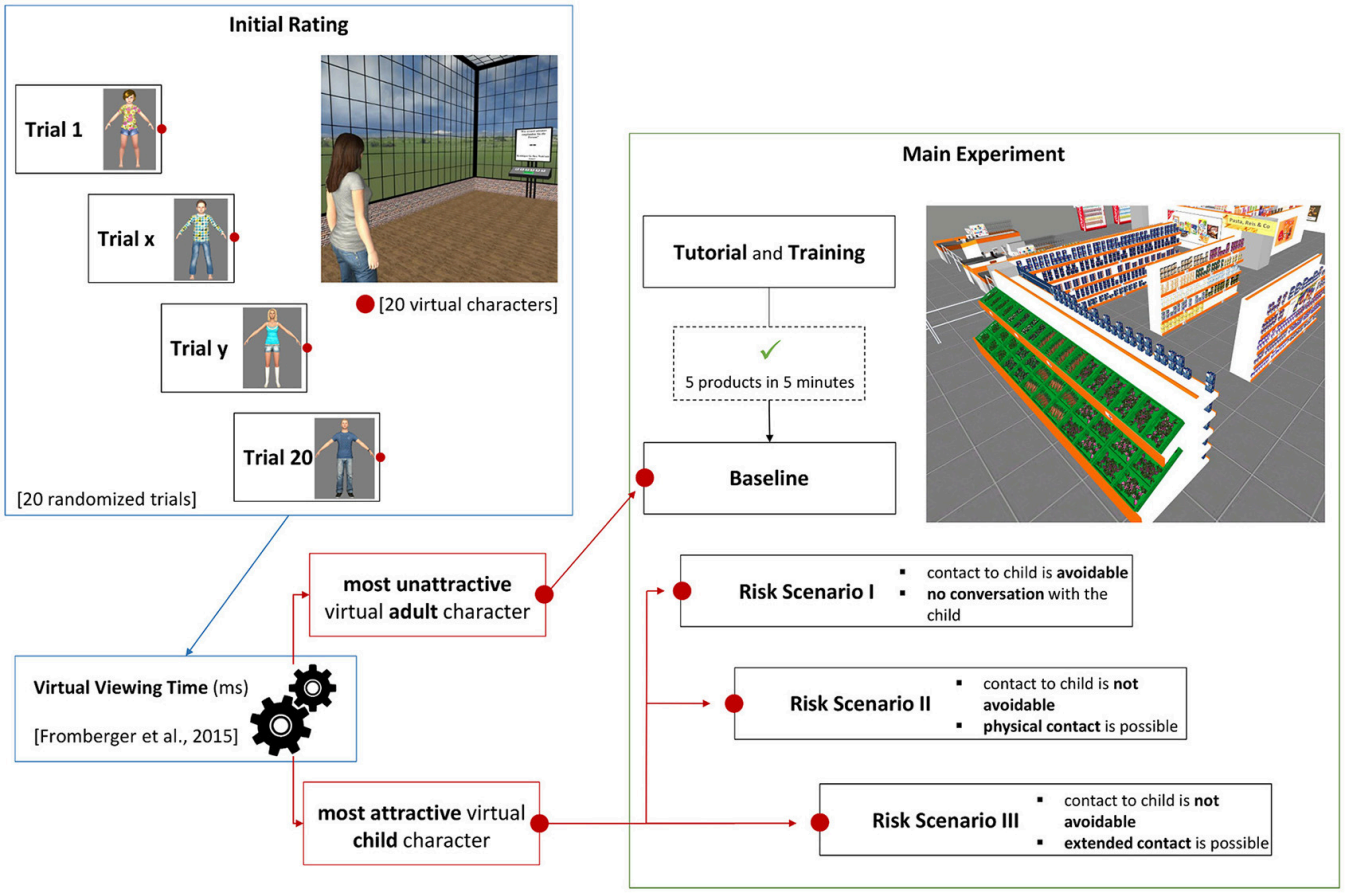
Experiment procedure. The experiment started with the initial rating. Goal of the initial rating was to identify the individual most unattractive virtual adult and most attractive virtual child character. In the main experiment phase, the participants learned the controlling of the virtual supermarket and the task in the tutorial and training. Afterwards, the participant was exposed to the most attractive virtual child character consecutively in three risk scenarios. The risk scenarios differed from each other with regard to their difficulty to avoid the contact to the virtual child character.

The experiment was divided into two phases, which took place on two different dates (see Figure 2). In the first phase, an initial rating took place in order to identify the most attractive virtual child character and the most unattractive virtual adult character for each individual participant (see Supplementary S1 and Supplementary S2 Initial Rating for further details). The main experiment phase had five parts: Tutorial and Training, a Baseline condition, and three virtual risk scenarios. Before each part, the participant was asked to fill in the pre-test of the Simulator Sickness Questionnaire (Kennedy et al., 1993, SSQ) and the Patient Rating Scale for Virtual Risk Scenarios (P-VRS). After each part, the participant was asked to fill in the post-test of the SSQ, the Igroup Presence Questionnaire (Schubert et al., 2001, IPQ), the Social Presence Questionnaire (Bailenson et al., 2005, SPQ), and the German VR Simulation Realism Scale (Poeschl and Doering, 2013, VRSRS). Between the different parts of the main experiment phase, the participant has the opportunity to rest.

#### 2.3.1. Tutorial and training

During the tutorial, the participant was instructed that his task is to buy five specific products (chocolate, Coca-Cola, pasta, milk, and coffee) in the virtual supermarket within 5 min. At the beginning, the participants stood in the entrance area in front of the virtual screen. The investigator explained the controlling of the VE and asked the participant to test the two different options to walk through the supermarket (per joystick or per pedes; see above). Then, the investigator guided the participant during his first virtual shopping trip. In order to ensure that all participants were at the same training level regarding the control of the VE, the participants trained in the VE until they were able to buy all five products within 5 min. During this training phase, the participant received no help from the investigator.

#### 2.3.2. Baseline condition

At the beginning of the baseline condition, the participants were told that the task to buy five products remained, but with no time restriction. They were further told, that a virtual character will make contact during the virtual shopping trip. Responses can be selected with the HUD. Based on the results of the initial rating, the virtual adult character with the shortest viewing time was used. Until the participant reached the candy shelves in the middle of the virtual supermarket (the participant had to buy chocolate), the virtual character walked through the supermarket at a predefined path. When the participant reached the candy shelves, the virtual character walked to the participant and asked, if the participant knew where he can find Coca-Cola bottles in the supermarket. During the interaction, the participant was not able to move. After the virtual character talked to the participant, the participant was able to choose among five different answers. Depending on the chosen answer, the virtual character reacted in different ways (see Supplementary S4). The baseline situation finished once the participant reached the entrance area.

#### 2.3.3. Risk scenarios

After finishing the baseline condition, the participant had to walk through three virtual risk scenarios in a fixed order. The task was the same as in the baseline condition. In contrast to the baseline condition, in the risk scenarios the participant was confronted with the most attractive virtual child character according to the results of the initial rating (the virtual child character with the longest viewing time). The same virtual child character was used in all three virtual risk scenarios. The virtual risk scenarios differed with regards to their difficulty to avoid a contact to the virtual child characters. In risk scenario one, the participant has the opportunity to avoid direct contact with the child at all. In risk scenario two and three, the participant was not able to avoid contact to the virtual child, but he could leave (or not) the situation immediately (see Supplementary S5–S7). In each risk scenario, the virtual child character walked through the supermarket at a pre-defined path until the participant reached a specific trigger area of the supermarket (risk scenario one and two: candy shelves; risk scenario three: cash-points). The path of the virtual child character was defined in order to ensure, that the participant could see the child before he was forced to get in contact with the child.

### 2.4. Measures

#### 2.4.1. Virtual reality: presence, realism, and simulator sickness

The *Simulator Sickness Questionnaire* (Kennedy et al., 1993, SSQ) is a self-report questionnaire to measure typical symptoms of simulator sickness. Its symptoms are similar to those of motion-induced sickness, but originate from elements of the visual display and visuo-vestibular interaction (Cobb et al., 1999; Kim et al., 2014). The questionnaire consists of 16 items based on a four-point Likert scale ranging from zero (the symptom is not existent) to three (very severe symptom). The SSQ consisted of three distinct symptom clusters (Oculomotor, Disorientation, and Nausea) and a total score. The total score measures the overall severity of simulator sickness.

The *Igroup Presence Questionnaire* (Schubert et al., 2001, IPQ) is a self-report questionnaire to measure the sense of presence in VR environments. It contains 14 items rated on a seven-point Likert scale ranging from zero to six. The IPQ contains three sub-scales that measure different components of presence: (1) the Spatial Presence sub-scale is related to the sense of physically being in the VE, (2) the Involvement sub-scale is meant to evaluate the attention devoted to the VE, and (3) the Realness sub-scale evaluates the sense of reality attributed to the VE. Additionally, the IPQ contains one general item that assesses the general “sense of being there,” and has a high loading on all three factors, with an especially strong loading on Spatial Presence.

The *Social Presence Questionnaire* (Bailenson et al., 2005, SPQ) is a self-report questionnaire to measure the sense of co-presence. Co-presence “reflects how user immersed in VR feel that virtual humans are really there, in the room, with them” (Bouchard et al., 2013, p. 62). Co-presence also reflects, if someone react to a virtual human as if it is a real human (Bailenson et al., 2005). The SPQ contains 10 items rated on a 7-point Likert scale ranging from zero (strongly disagree) to six (strongly agree). One factor of the SPQ measures the perceived co-presence. The perceived co-presence reflects how a user has the feeling, that a virtual human was really there in the VE.

The *German VR Simulation Realism Scale* (Poeschl and Doering, 2013, VRSRS) is a self-report questionnaire to measure the simulation realism. It consists of 14 items based on a 5-point Likert scale ranging from one (“I do not agree at all”) to five (“I fully agree”). Factor analysis identified four distinct factors [scene realism, audience behavior, audience appearance, and sound realism; Poeschl and Doering (2013)]. Scene realism measures the naturalism of visual cues, colors, three-dimensionality, and realistic proportions of the VE. Audience behavior measures the authenticity of postures, gestures, and facial expressions of virtual characters within the VE. The authenticity of virtual humans in general and the adequateness of the outfit of the virtual characters is subsumed under the factor audience appearance. Sound realism is a single item measure to describe the realism of the sound in general, e.g., if the sound corresponds to the VE.

#### 2.4.2. Virtual risk situations: behavior in the virtual environment

During each virtual scenario, the participant has to interact with a virtual character (baseline scenario, risk scenario two, and risk scenario three) or to decide how to react, upon seeing a child character at the candy shelves (risk scenario one). All these interactions were provided in mixed modalities: the virtual character talked to the participant and the participant could choose predefined answers or behaviors, which were presented via a HUD. The number of possible choices depended on the scenario and the choice the participant chose first (see Supplementary S5–S7 for an overview of possible interaction sequences in the different scenarios). There were a maximum of two interaction levels for baseline scenario, risk scenario two, and risk scenario three. In risk scenario I, only one interaction level was provided. All choices were categorized into *approach behavior* and *avoidance behavior*. Approach behavior was defined as every predefined answer in which the participant (1) could get in contact or tries to get in contact with a virtual character, (2) could touch or tries to touch the virtual character, (3) tries to extend the interaction sequence, or (4) tries to reduce the distance to the virtual character. Avoidance behavior was defined as every predefined answer, in which the participant (1) did not react to the virtual character or (2) tries to leave the situation. Supplementary S3–S5 show the categorization of each predefined answer in approach or avoidance behavior. Additionally, in order to compare the answer behavior with coping strategies learned during therapy (see the following section and Tables 2, 3), for some analyses (see Data analyses for more details) the approach behavior was further divided in approach behavior with physical contact or without physical contact.

**Table 2:** Items of the therapist rating scale for virtual risk scenarios (T-VRS).

**Item name (abbreviation)**	**Item content**	**Answer options**	**Target situation**
TherapistPredictionRecognition	“Is the patient able to recognize relevant risk situations?”	Yes / No	I, II, and III
TherapistPredictionAvoid	“Is the patient able to avoid contact to children?”	Yes / No	I
TherapistPredictionUnavoid	“Has the patient a sufficient control of behavior in order to show adequate coping strategies in situations in which the contact to children is unavoidable?	Yes / No	II, III
TherapistFocusAvoid	“It was in the focus of the therapy, that - in order to avoid a sexual assault against a child - …”	The patient should avoid any contact to children. The patient can have contacts with children, but he should not touch children. It does not matter if the patient has contact to a child. This aspect was not yet in the focus of the therapy.	I
TherapistFocusUnavoid	“It was in the focus of the therapy, that - in order to avoid a sexual assault against a child in a situation in which it is not possible to avoid the contact - …”	The patient should immediately break tie and leave the situation. The patient can stay in contact, but should avoid touching the child. It is no risk for the patient to be in contact with children. This aspect was not yet in the focus of the therapy.	II, III

**Table 3:** The items of the patient rating scale for virtual risk situations (P-VRS).

**Item name (abbreviation)**	**Item content**	**Answer options**	**Target situation**
PatientBeliefAvoid	“I belief, that - in order to avoid a sexual assault against a child - …”	I should avoid any contact to children. I can have contacts with children, but I should not touch children. It does not matter if I have contact to a child.	I
PatientBeliefUnavoid	“I belief, that - in order to avoid a sexual assault against a child in a situation in which it is not possible to avoid the contact - …”	I should immediately break tie and leave the situation. I can stay in contact, but should avoid touching the child. It is no risk for me to be in contact with children.	II, III
PatientFocusAvoid	“During therapy I have learned, that - in order to avoid a sexual assault against a child - …”	I should avoid any contact to children. I can have contacts with children, but I should not touch children. It does not matter if I have contact to a child. This aspect was not yet in the focus of the therapy.	I
PatientFocusUnavoid	“During therapy I have learned, that - in order to avoid a sexual assault against a child in a situation in which it is not possible to avoid the contact - …”	I should immediately break tie and leave the situation. I can stay in contact, but should avoid touching the child. It is no risk for me to be in contact with children. This aspect was not yet in the focus of the therapy.	II, III

The *Therapist Rating Scale for Virtual Risk Scenarios* (T-VRS) was developed in order to assess coping strategies focused on during therapy by the therapist and the prediction of therapists with regards to the ability of the participant to perform learned coping strategies. Table 2 describes all items of the T-VRS in detail. It consists of two dichotomous items, which were explicitly linked to risk situations comparable to the virtual risk situations and asks for the prediction of the therapists with regards to the ability of the SOC to cope with risk situations. Note that all therapists had walked through the virtual risk scenarios before filling in the T-VRS. One item corresponds to risk situation I in which the contact with a child was avoidable (“TherapistPredictionAvoid”) and one item to risk situation II and III in which contact was not avoidable (“TherapistPredictionUnavoid”). Two further items (‘TherapistFocusAvoid” for risk situation I, “TherapistFocusUnavoid” for risk situation II and III) asking for the coping strategies which the therapist focused on during therapy. Another dichotomous item (“TherapistPredictionRecognition”) asked about the ability to recognize risk situations as “risky.”

The *Patient Rating Scale for Virtual Risk Scenarios* (P-VRS) was developed in order to assess the subject beliefs of SOCs about the correct behavior in risk situations comparable to the virtual risk scenarios and to assess coping strategies patients have learned during therapy. This aspect was assessed with two items, one item for risk situation I in which contact with a child was avoidable (“PatientBeliefAvoid”) and one item for risk situation II and III in which contact with a child was not avoidable (“PatientBeliefUnavoid”). With two further items (“PatientFocusAvoid” for risk situation I; “PatientFocusUnavoid” for risk situation II and III), the P-VRS assess the coping strategies SOCs stated they have learned during the therapy. Table 3 describes all items of the P-VRS in detail. Note that the T-VRS and the P-VRS were only applied to SOCs.

### 2.5. Data analysis

Data analysis was performed with the statistic software R (R Core Team, 2016, Version 3.2.2). In order to evaluate the acceptance of the virtual risk scenarios between the two groups (SOCs vs. NOCs), a Welch *t*-test was performed for the factor Copresence of the SPQ, the factor “general item” of the IPQ and all factors of the VRSRS in each experimental condition (baseline, scenario one, scenario two, scenario three). In order to identify changes with regard to simulator sickness symptoms, a paired *t*-test (pre vs. post) was performed for the General factor of the SSQ separately for each participant group and experimental condition. Due to low sample size, the effect-size Cohen's *d* was used to interpret the results and not the *p*-value.

The frequency of approach and avoidance behavior of NOCs and SOCs was compared for each scenario with Fisher's Exact tests. The effect size Cramer' V was used to interpret the results due to the small sample size. Because of the small sample size, we decided not to perform further statistical analysis within the group of SOCs or NOCs with regard to their behavior. Thus, only descriptive statistics are provided.

In order to evaluate, if SOCs behavior was in concordance with their subject belief about correct behavior, the frequencies of congruent and not congruent behavior with the respective items of P-VRS were calculated. For risk situation I, the congruence with the P-VRS item “PatientbeliefAvoid” and for risk situation II and III, the congruence with the P-VRS item “PatientBeliefUnavoid” was calculated. In order to evaluate if SOCs behavior was in concordance with coping strategies SOCs have stated that they have learned in therapy, the frequencies of congruent and not congruent behavior with the respective items of P-VRS were calculated (item “PatientFocusAvoid” for scenario I and item “PatientFocusUnavoid” of the P-VRS for scenarios II and III). To evaluate the concordance of SOCs behavior with therapists' focus of the therapy, the frequencies of congruent and not congruent behavior of SOCs with the respective T-VRS items were calculated (item “TherapistFocusAvoid” for scenario I and item “TherapistFocusUnavoid” of the T-VRS for scenarios II and III).

Furthermore, the frequency of correct and incorrect predictions of SOC's behavior in virtual risk situations by their therapists (*n* = 3) was calculated (based on the item “TherapistPredictionAvoid” for scenario I and item “TherapistPredictionUnavoid” of the T-VRS for scenario II and II). Here, the focus of the therapy by the therapists (item “TherapistFocusAvoid” for scenario I and item “TherapistFocusUnavoid” of the T-VRS for scenarios II and III) was considered. For example, if a SOC showed approach behavior without physical contact in risk scenario II and the therapists' focus of the therapy was, that contact to children can be maintained and only physical contact has to be avoided (as stated by the therapist in item “TherapistFocusUnavoid” of the T-VRS). If additionally the therapist predicted that the patient would be able to cope with such situations (item “TherapistPredictionUnavoid” of the T-VRS), then this was considered as a correct prediction.

## 3. Results

### 3.1. Virtual reality related measures

#### 3.1.1. Presence (igroup presence questionnaire, IPQ)

As shown in Table 4, the reported subjective feeling of presence was at a high level in each experimental condition. The statistical analysis of the General item (“sense of being there”) revealed differences between SOCs and NOCs with only small effect sizes [baseline: *t*_(10.72)_ = −0.601, *p* = 0.501, 95% CI (−1.99, 1.13), *d* = 0.337; scenario one: *t*_(10.99)_ = −0.690, *p* = 0.505, 95% CI (−1.70, 0.888), *d* = 0.381; scenario two: *t*_(8.43)_ = 0.318, *p* = 0.758, 95% CI (−1.62, 2.14), *d* = 0.180; scenario three: *t*_(9.36)_ = −0.548, *p* = 0.596, 95% CI [−2.06, 1.26], *d* = 0.308]. Thus, independent of the experimental condition there were if at all only small differences with regards to the feeling of presence between the participant groups. Furthermore, a reliability analysis revealed an overall acceptable internal consistency of Cronbach's α = 0.74.

**Table 4:** Means and SDs of the Igroup Presence Questionnaire (IPQ) subscale general factor (“sense of being there”), of the Social Presence Questionnaire (SPQ) subscale Copresence, and of the Simulator Sickness Questionnaire (SSQ) subscale General factor.

**Group**	**Condition**	**Presence**	**Co-presence**	**Simulator sickness (pre)**	**Simulator sickness (post)**
		***M* (*SD*)**	***M* (*SD*)**	***M* (*SD*)**	***M* (*SD*)**
**Non sexual offenders against children**	Baseline	4.57 (1.27)	3.14 (1.07)	5.00 (10.68)	4.14 (8.30)
	Scenario I	4.43 (1.13)	3.14 (1.46)	3.00 (6.71)	6.71 (12.71)
	Scenario II	4.43 (1.13)	3.29 (0.95)	7.29 (18.00)	9.00 (16.32)
	Scenario III	4.43 (1.13)	3.14 (0.90)	3.57 (8.20)	5.71 (9.83)
**sexual offenders against children**	Baseline	5.00 (1.26)	2.50 (1.52)	4.17 (8.82)	4.83 (11.84)
	Scenario I	4.83 (.98)	2.17 (1.60)	1.83 (4.49)	2.20 (4.92)
	Scenario II	4.20 (1.72)	2.67 (1.63)	2.20 (4.92)	3.00 (4.82)
	Scenario III	4.83 (1.47)	2.83 (2.04)	1.83 (4.49)	1.83 (4.49)

*The General factor of the SSQ was measured before and after each virtual scenario. The IPQ and PQ subscales range from zero to six. The SSQ subscale Total score ranges from zero to 235.62*.

#### 3.1.2. Copresence (social presence questionnaire, SPQ)

Table 4 shows the means and SDs for the SPQ subscale Copresence. As one can see, the subjective Copresence was at a medium level in all conditions and both participant groups. The statistical analysis of the factor Copresence revealed differences between the two participant groups with only small effect sizes in the baseline condition [*t*_(8.83)_ = 0.870, *p* = 0.408, 95% CI (−1.03, 2.32), *d* = 0.490), scenario two (*t*_(6.65)_ = 0.817, *p* = 0.438, 95% CI (−1.14, 2.37), *d* = 0.463], and scenario three [*t*_(6.65)_ = 0.344, *p* = 0.742, 95% CI (−1.84, 2.46), *d* = 0.196]. A difference with a medium effect size was observed in scenario one [*t*_(10.72)_ = 1.14, *p* = 0.296, 95% CI (−0.924, 2.88), *d* = 0.636]. Internal consistency for the factor Copresence was poor (Cronbach's α = 0.55).

#### 3.1.3. Simulator sickness (simulator sickness questionnaire, SSQ)

As shown in Table 4, the reported severity of simulator sickness symptoms were at a low level after each experimental condition in both participant groups. Paired t-tests between the pre and post value of the Total Score of the SSQ revealed differences with only small effect sizes in SOCs [baseline: *t*_(5)_ = −0.491, *p* = 0.644, 95% CI (−4.16, 2.82), *d* = 0.200; scenario one: *t*_(4)_ = −1.00, *p* = 0.374, 95% CI (−8.31, 3.91), *d* = 0.447; scenario two: statistical analysis is not possible since pre and post values were identical for all participants; scenario three: statistical analysis is not possible since pre and post values were identical for all participants]. In addition, NOCs showed only differences with only small effect sizes in the baseline condition [*t*_(6)_ = 0.670, *p* = 0.528, 95% CI (−2.28, 3.99), *d* = 0.253], scenario two [*t*_(6)_ = −0.563, *p* = 0.594, 95% CI (−9.16, 5.74), *d* = 0.213], and scenario three [*t*_(6)_ = −1.00, *p* = 0.356, 95% CI (−7.39, 3.10), *d* = 0.378]. Only in scenario one, NOCs showed a difference between pre and post symptoms with a medium effect size [*t*_(6)_ = −1.52, *p* = 0.178, 95% CI (−9.68, 2.25), *d* = 0.576]. The SSQ provided good internal consistency (Cronbach's α = 0.88). Thus, one can assume that there was no significant increase of simulator sickness symptoms during the experimental conditions. That holds true for both participant groups, except NOCs in scenario one. Bearing the maximum value of 235.62 of the Total Score in mind, an increased Total Score of 6.17 after scenario one in NOCs cannot be interpreted as a rise in simulator sickness symptoms.

#### 3.1.4. Realism (German VR simulation realism scale, VRSRS)

Means and SDs for Scene Realism, Audience Behavior and Sound Realism of the VRSRS are shown as a function of Group and Condition in Table 5. Audience Appearance could not be analyzed due to too many missing values. Welch tests for the factor Scene Realism revealed differences between the two groups in all experimental conditions with low to medium effect sizes [baseline: *t*_(10.00)_ = −0.576, *p* = 0.578, 95% CI (−4.76, 2.80), *d* = 0.322; scenario one: *t*_(10.76)_ = −0.855, *p* = 0.411, 95% CI (−5.29, 2.33), *d* = 0.475; scenario two: *t*_(10.45)_ = −1.13, *p* = 0.283, 95% CI (−5.49, 1.78]), *d* = 0.631; scenario three: *t*_(11.00)_ = −1.19, *p* = .596, 95% CI (−5.29, 1.58), *d* = 0.658]. The two groups differed with regards to the factor Audience Behavior at a medium effect size level in the baseline condition [*t*_(11.00)_ = −0.680, *p* = 0.511, 95% CI (−3.53. 1.87), *d* = 0.376]. In scenario one [*t*_(9.39)_ = −3.06, *p* = 0.013, 95% CI (−5.32, −0.820), *d* = 1.67] and in scenario three [*t*_(10.77)_ = −1.54, *p* = 0.154, 95% CI (−5.34, 0.958), *d* = 0.844] the two groups differed by a high effect size. At scenario two only a group difference of medium effect size emerged [*t*_(10.97)_ = −1.31, *p* = 0.218, 95% CI (−4.60, 1.17), *d* = 0.721]. Except the baseline condition with a high effect size level [*t*_(6)_ = 1.55, *p* = 0.172, 95% CI (−0.166, 0.737), *d* = 0.828], Welch tests revealed group differences with regards to Sound Realism at a medium effect size level in all scenarios [scenario one: *t*_(6)_ = 1.00, *p* = 0.356, 95% CI (−0.207, 0.492), *d* = 0.535; scenario two: *t*_(6)_ = 1.00, *p* = 0.356, 95% CI (−0.207, 0.492), *d* = 0.535; scenario three: *t*_(6)_ = 1.00, *p* = 0.356, 95% CI (−0.207, 0.492), *d* = 0.535]. The VRSRS provided a good general reliability (Cronbach's α = 0.85).

**Table 5:** Means and SDs of the German VR Simulation Realism Scale (VRSRS) subscales as a function of Group and Condition.

**Group**	**Condition**	**Scene realism**	**Audience behavior**	**Sound realism**
		***M* (*SD*)**	***M* (*SD*)**	***M* (*SD*)**
**Non sexual offenders against children**	Baseline	18.86 (2.79)	14.00 (2.38)	3.29 (0.49)
	Scenario I	18.86 (3.13)	13.43 (2.97)	3.14 (0.38)
	Scenario II	19.14 (2.85)	14.29 (2.63)	3.14 (0.38)
	Scenario III	19.14 (3.02)	15.14 (2.73)	3.14 (0.38)
**Sexual offenders against children**	Baseline	19.83 (3.25)	14.83 (2.04)	3.00 (0.00)
	Scenario I	20.33 (3.08)	16.50 (1.22)	3.00 (0.00)
	Scenario II	21.00 (3.03)	16.00 (2.10)	3.00 (0.00)
	Scenario III	21.00 (2.61)	17.33 (2.16)	3.00 (0.00)

*Scene realism ranges from zero to 25, Audience behavior from zero to 20, and Sound realism from zero to 6*.

### 3.2. Behavior in the virtual environment

#### 3.2.1. Comparison between sexual offenders against children and non-offender controls

Table 6 shows the frequency of approach and avoidance behavior in the baseline condition. In the baseline scenario, one SOC (17%) showed approach behavior at both interaction levels, one SOC (17%) showed approach behavior at the first interaction level and avoidance behavior at the second interaction level, and four SOCs (67%) showed avoidance behavior. In contrast, six NOCs (86%) showed approach behavior at both interaction levels and one NOC (14%) showed avoidance behavior. The behavior of SOCs and healthy controls differed significantly in the baseline scenario with a large effect size (*Fisher's Exact Test: p* = 0.041, *V* = 0.700).

**Table 6:** χ^2^-table for the approach and avoidance behavior of SOCs and NOCs in the baseline condition.

**Group**		**Behavior**				**Cramer's V**
		**Approach - approach**	**Approach - avoidance**	**Avoidance**		
		***N* = 7**	***N* = 1**	***N* = 5**		
SOC	*N* = 6	17% (1)	17% (1)	67% (4)	100%	*V* = 0.700
NOC	*N* = 7	86% (6)	0% (0)	14% (1)	100%	

*In the baseline scenario were two possible interaction levels. Thus the participant was able to choose approach behavior at the first and second interaction level (approach-approach), approach behavior at the first interaction level and avoidance behavior at the second interaction level (approach-avoidance), or avoidance behavior at the first interaction level (avoidance; the scenario stops afterwards). The effect size Cramer's V for the difference between the two groups with regard to their behavior is reported*.

In risk scenario one (Table 7), five SOCs (83%) showed approach behavior and one SOC (17%) demonstrated avoidance behavior. All healthy controls (100%) showed approach behavior. There was a difference between the behavior of SOCs and healthy controls at a medium effect size level (*Fisher's Exact Test: p* = 0.462, *V* = 0.311).

**Table 7:** χ^2^-table for the approach and avoidance behavior of SOCs and NOCs in risk scenario one.

**Group**		**Behavior**			**Cramer's V**
		**Approach**	**Avoidance**		
		***N* = 12**	***N* = 1**		
SOC	*N* = 6	83% (5)	17% (1)	100%	*V* = 0.311
NOC	*N* = 7	100% (7)	0% (0)	100%	

*In risk scenario one, only one interaction level with avoidance or approach behavior was possible. The effect size Cramer's V for the difference between the two groups with regard to their behavior is reported*.

During virtual risk scenario two (Table 8), one SOC (17%) showed approach behavior at both interaction levels, four SOCs (67%) demonstrated approach behavior at the first interaction level followed by avoidance behavior at the second interaction level, and one SOC showed avoidance behavior (17%). In contrast, two healthy controls (29%) showed approach behavior at both interaction levels, and five healthy controls (71%) showed approach behavior at the first interaction level and avoidance behavior at the second interaction level. No healthy control participant showed avoidance behavior. There was a difference between the behavior of SOCs and healthy controls in risk situation two at a medium effect size level (*Fisher's Exact Test: p* = 1.00, *V* = 0.326).

**Table 8:** χ^2^-table for the approach and avoidance behavior of SOCs and NOCs in trisk scenario two.

**Group**		**Behavior**				**Cramer's V**
		**Approach - approach**	**Approach - avoidance**	**Avoidance**		
		***N* = 3**	***N* = 9**	***N* = 1**		
SOC	*N* = 6	17% (1)	67% (4)	17% (1)	100%	*V* = 0.326
NOC	*N* = 7	29% (2)	71% (5)	0% (0)	100%	

*In risk scenario two were two possible interaction levels. Thus the participant was able to choose approach behavior at the first and second interaction level (approach-approach), approach behavior at the first interaction level and avoidance behavior at the second interaction level (approach-avoidance), or avoidance behavior at the first interaction level (avoidance; the scenario stops afterwards). The effect size Cramer's V for the difference between the two groups with regard to their behavior is reported*.

During virtual risk scenario three (Table 9), three SOCs (50%) showed approach behavior at both interaction levels, one SOC (17%) showed approach behavior at the first interaction level and avoidance behavior at the second interaction level, and two SOC (33%) demonstrated avoidance behavior. In contrast, all healthy controls (100%) demonstrated approach behavior at both interaction levels. The difference between the behavior of SOCs and healthy controls in risk situation three was at a large effect size level (*Fisher's Exact Test: p* = 0.070, *V* = 0.439).

**Table 9:** χ^2^-table for the approach and avoidance behavior of SOCs and NOCs in risk scenario three.

**Group**		**Behavior**				**Cramer's V**
		**Approach - approach**	**Approach - avoidance**	**Avoidance**		
		***N* = 10**	***N* = 1**	***N* = 2**		
SOC	*N* = 6	50% (3)	17% (1)	33% (2)	100%	*V* = 0.439
NOC	*N* = 7	100% (7)	0% (0)	0% (0)	100%	

*In risk scenario three were two possible interaction levels. Thus the participant was able to choose approach behavior at the first and second interaction level (approach-approach), approach behavior at the first interaction level and avoidance behavior at the second interaction level (approach-avoidance), or avoidance behavior at the first interaction level (avoidance; the scenario stops afterwards). The effect size Cramer's V for the difference between the two groups with regard to their behavior is reported*.

#### 3.2.2. Congruence between behavior and belief of patients about correct behavior (P-VRS)

Summarized over all three risk situations, in 89% (*n* = 16) of all cases, SOCs showed a behavior which was not congruent to the belief of the SOC about correct behavior (see Table 10 for a full 2 × 2 contingency table). Thus, only in 11% (*n* = 2) of all cases SOCs behaved in accordance to their own belief how they should behave in comparable situations. Note that only approach behavior with the aim to touch a child was categorized as in-congruent to the statement “…can stay in contact, but should avoid touching the child.”

**Table 10:** 2 × 2 contingency table of the congruence between SOC's behavior and belief about correct behavior in comparable situations.

		**Behavior**	
		**Avoidance**	**Approach**
Belief	Avoidance	11% (2) [TP]	78% (14) [FP]
	Approach	11% (2) [FN]	0% (0) [TN]

*TP, True Positive; FP, False Positive; TN, True Negative; FN, False Negative*.

#### 3.2.3. Congruence between behavior and learned coping strategies (P-VRS)

Summarized over all three risk situations (and excluding all cases in which coping strategies were not yet the focus of the therapy, *n* = 5), in 62% (*n* = 8) of all cases SOCs showed a behavior which was not congruent with the coping strategy they stated that they have learned during therapy (see Table 11 for a full 2 × 2 contingency table). Only in 38% (*n* = 5) of all cases SOCs behaved in accordance with the coping strategy they stated that they have learned during therapy.

**Table 11:** 2 × 2 contingency table of the congruence between SOC's behavior and in therapy learned coping strategies for comparable situations.

		**Behavior**	
		**Avoidance**	**Approach**
Learned strategy	Avoidance	31% (4) [TP]	62% (8) [FP]
	Approach	0% (0) [FN]	8% (1) [TN]

*All cases are excluded in which coping strategies were not yet learned or in the focus of the therapy (details are given in the text)*.

*TP, True Positive; FP, False Positive; TN, True Negative, FN, False Negative*.

#### 3.2.4. Congruence between behavior and coping strategies focused in therapy by the therapist (T-VRS)

Summarized over all three risk situations (and excluding all cases in which coping strategies were not yet in the focus of the therapy, *n* = 6), in 50% (*n* = 6) of all cases SOCs showed a behavior which was not congruent with the coping strategy that was the focus of the therapy (see Table 12 for a full 2 × 2 contingency table). Note, that only approach behavior with the aim to touch a child was categorized as in-congruent to the statement “…can stay in contact, but should avoid touching the child.”

**Table 12:** 2 × 2 contingency table of the congruence between SOC's behavior and coping strategies focused during therapy as stated by the therapists.

		**Behavior**	
		**Avoidance**	**Approach**
Focused strategy	Avoidance	8% (1) [TP]	25% (3) [FP]
	Approach	25% (3) [FN]	42% (5) [TN]

*All cases are excluded in which coping strategies were not yet learned or in the focus of the therapy (details are given in the text)*.

*TP, True Positive; FP, False Positive; TN, True Negative, FN, False Negative*.

#### 3.2.5. Congruence between behavior and therapist's prediction (T-VRS)

Summarized over all three risk situations (excluding all cases in which coping strategies were not yet in the focus of the therapy, *n* = 6), in 75% (*n* = 9) of all cases therapists predicted the behavior of the SOCs based on their therapy content correctly. Thus, in 25% (*n* = 3) of all cases therapists made a false prediction. See Table 13 for a full 2 × 2 contingency table of therapists' predictions.

**Table 13:** 2 × 2 contingency table of therapist's predictions of the SOC's behavior in comparable situations.

		**Behavior**	
		**Avoidance**	**Approach**
Therapist's prediction	avoidance	75% (9) [TP]	25% (3) [FP]
	approach	0% (0) [FN]	0% (0) [TN]

*All cases are excluded in which coping strategies were not yet learned or in the focus of the therapy (details are given in the text)*.

*TP, True Positive; FP, False Positive; TN, True Negative, FN, False Negative*.

## 4. Discussion

In the current feasibility study, the usability of behavioral monitoring of SOCs in virtual risk situations for clinical risk management was evaluated. Three risk scenarios, which match possible real high-risk situations during a first unsupervised walk out of the secured ward to town, were developed. The participants had to buy specific products in a virtual supermarket, during which they were confronted with a virtual child. The behavior of the participants was assessed by the participant's answers during the interaction with the virtual child. The predefined answer options were designed following the RP approach teaching SOCs coping skills to avoid risk situations or to cope with unavoidable risk situations. One aim of the study was to evaluate if virtual risk scenarios provide a high feeling of presence and co-presence, low symptoms of simulator sickness and high subjective feelings of realism for forensic inpatients who had sexually abused a child. Main aim of the study was to evaluate if the confrontation of SOCs with virtual risk situations can provide additional information for the decision about unsupervised privileges outside the secured ward of a forensic psychiatry beyond behavioral monitoring in controlled environments and traditional risk assessment tools.

### 4.1. Are virtual environments usable for forensic inpatients who have sexually abused a child?

The results with regards to presence, co-presence, realism, and simulator sickness showed that VEs are well suitable for forensic inpatients who sexually abused a child: Results demonstrated that differences between SOCs and NOCs with regards to their subjective feeling of presence are at a small effect size level. Thus, both groups had a high feeling of being in the VE. There was also only a difference at a small effect size level between the groups with regards to the subjective feeling of co-presence in all experimental conditions except scenario one. In scenario one, the groups differed by a medium effect size level. Here, NOCs had a somewhat greater feeling that the virtual characters were really there in the VE. The subjective realism of the virtual scenes as well as the realism of the behavior of virtual characters and the sound was high for both participant groups. Thus, the virtual scenarios were noticed as realistic with regards to three-dimensionality, proportions and colors. Furthermore, the postures, gestures, voices, and facial expressions of the virtual characters were regarded as authentic. This shows that the virtual characters have been designed authentic and have been well animated, even for SOCs. The two groups differed in their subjective realism ratings, especially in the rating of the realism of the behavior of the virtual characters partly at a high effect size level. SOCs rated the behavior as more realistic than NOCs. Furthermore, both groups didn't suffer from simulator sickness symptoms. Simulator sickness is one of the main problems with high-immersive VEs and most important for the usability of VEs (Kennedy et al., 1993). It was shown that simulator sickness can be reduced by using motion capturing instead of more traditional game controllers for navigation (Llorach et al., 2014). During risk scenarios, both techniques were combined, which could explain the low frequency of simulator sickness symptoms. In summary and facing the age difference between NOCs and SOCs as well as the differences with regards to the education level and the accessibility of e.g., 3D cinemas or state-of-the-art computer games, these results are promising. It shows that the new VR technology seems to be well accepted by forensic inpatients, and seems to be able to provide a high ecological valid environment for forensic inpatients, also for elderly patients.

### 4.2. Can behavioral monitoring of sexual offenders against children in virtual risk situations provide useful information for risk management?

It was hypothesized that behavioral monitoring of SOCs in virtual risk situations could provide information about the ability of SOCs to show adequate coping strategies in risk situations. With some restriction, the results support this hypothesis: Firstly, results demonstrated that the approach-avoidance behavior of SOCs and NOCs only differed in the baseline scenario and risk scenario three at a high effect size level. In the baseline scenario, SOCs showed more avoidance behavior than NOCs. In the risk situations one and two, NOCs showed approach behavior more frequently as SOCs (the two groups differed by a medium effect size level). In risk scenario three, all NOCs showed approach behavior, but also 50% of SOCs. The scenarios were conceptualized as help situations and it is not surprising that NOCs choose to help the children. In contrast, SOCs should have learned during the RP based treatment to avoid getting in touch with children. Thus, behavioral monitoring in virtual risk situations could provide information about the ability of SOCs to show adequate coping strategies. At first glance, SOCs chose inadequate coping behaviors in the majority of all cases.

In order to arrange the behavior of SOCs in the virtual risk situations correctly, it is necessary to consider what SOCs have learned during therapy, what therapists focused on during therapy, and what they consider the correct behavior is in such situations. Therefore, the congruence between the knowledge of the SOCs about correct behaviors in comparable situations and monitored behavior during virtual risk situations was analyzed. Only in 11% of all cases, the SOCs behaved in accordance with their own knowledge. In addition, there was a low congruence between learned coping strategies (as stated by the patients) and the shown behavior in risk situations (38%). Most congruence was seen between SOC's behavior and coping strategies therapists focused on during therapy, however only in 50% of all cases. Thus, it seems that SOCs behaved half of the time in correspondence with the coping skills which therapists focused on. Bearing that in mind, the lack of a significant difference between SOCs and NOCs in risk scenario II and III seems to be the result of using coping skills the patients learned during therapy. On the other side, behavioral monitoring of SOCs in virtual risk situations demonstrated in 50% of all cases, that SOCs were not able to transfer the coping skills therapists focused on in therapy or the coping skills they have learned during therapy. Possibly, this is a result of low self-regulation abilities of the SOCs. Thus, behavioral monitoring of SOCs in virtual risk situations seems to provide information about the ability of SOCs to transfer the (at a cognitive level) learned coping strategies to the behavioral level. This information can be important for decisions about unsupervised privileges and for risk management in general. For example, Marques et al. (2005) reported, that sex offenders treated with the RP approach and who successfully learned coping skills had significantly lower recidivism rates (13.5%) than sex offenders who did not learned coping strategies successful (27.2%). This difference with regards to re-offense rates was more dominant in the group of high-risk sex offenders and most dominant in the group of child molesters. Only 9.3% of child molesters who had successfully learned coping skills re-offended, but 31.3% of child molesters who did not learned coping skills successfully. These results underline the importance to evaluate if coping skills are successfully learned during therapy before permission of unsupervised privileges. Therefore, behavioral monitoring of SOCs in virtual risk situations seems to provide necessary information for the decision about unsupervised privileges.

The difference between SOCs and NOCs in risk scenario I, in which a contact was avoidable, at a medium effect size level seems to be the result of not following the suggestions of the therapists for comparable situations. Also in scenario I, the most frequent discrepancy between behavior and during therapy focused coping skills occurred. One possible explanation for this result could be the existence of an order effect. The presentation order of the risk scenarios was fixed, beginning with risk scenario one and ending with risk scenario three. Thus, it could be possible that SOCs did not recognize the situations as risk situations until they walked through the first situation. From a risk management point of view, it is important that SOCs recognize situations as risk situations as soon as they occur. The ability to recognize situations as risk situations is one important skill, which should be learned during RP based treatment (Laws et al., 2000).

The lack of congruence between the behavior and the knowledge of the SOCs about correct behavior as well as the lack of congruence between the learned behavior and shown behavior during risk situations is striking. Moreover, there is an obvious mismatch between the learned behavior, SOCs stated and the focus of the therapy as reported by the therapists. A possible explanation could be that SOCs answered these questions in a social desirable manner. It is well known that SOCs show tendencies to answer such questions in a social desirable manner (O'Donohue et al., 2000). It is possible that SOCs indicated what they have heard during therapy, without being convinced that this is adequate behavior or without having internalized the learned coping strategies.

One can further argue that sexual offenders against children with average intelligence may be able to adapt their behavior in the expected direction, since they know that their behavior is observed during immersion. Possibly, the lack of congruence between the behavior and the knowledge of the SOCs about correct behavior is the result of intelligence deficits or emotional or introspective shortcomings within the studied sample of sexual offenders against children. These shortcomings may also be the reason for not succeeding in psychotherapy. Future studies have to consider this before VR can be used in clinical settings.

### 4.3. Can therapists predict behavior of sexual offenders against children in virtual risk situations?

Therpists predicted the behavior of SOCs in virtual risk situations in 75% of all cases correctly. Assuming, that therapists made their predictions based on risk assessment tools as well as behavioral monitoring in controlled environments, the confrontation of SOCs with virtual risk scenarios seems to provide at first glance no additional information to traditional risk management. Nevertheless, for the first time, virtual risk scenarios enable the therapist to evaluate their predictions and the ability of SOCs to behave in correspondence to the coping strategies, which were focused on during therapy. This seems to be an important aspect: to be able to evaluate the therapeutic process and to be able to predict the outcome of the treatment.

On the other hand, in 25% of all cases, therapists were not able to predict the behavior of their patients correctly. This was due to not applying adequate behavior as expected (false positives; see Table 13). For these cases, behavioral monitoring in virtual risk situations can provide essential new information useful for risk management decisions. It can also lead to re-evaluating the made predictions and the focus of therapy by the therapist.

### 4.4. Does behavior in virtual risk situations reflect behavior in real-life?

Behavioral monitoring in virtual risk situations can only be useful when the behavior of SOCs in virtual risk situations reflects the behavior in real risk situations. Up to now, no empirical data exists, if the behavior of SOCs in VEs is a valid predictor for the behavior of SOCs in real situations. However, VR studies in other contexts have already shown that behavior learned in VEs can be transferred to real situations. In a current meta-analyses, Morina et al. (2015) demonstrated, based on the data of 14 clinical trials of VR exposure therapy for specific phobias, that participants performed significantly better in behavioral (real) assessments than before the VR treatment and better than patients on waiting-lists. Furthermore, the performance of patients with VR treatment did not differ significantly from the performance of patients treated with exposure *in vivo*. The authors concluded, that VR treatment seems to result in significant behavioral changes in real-life at least for patients suffering from specific phobias. In the already mentioned study by Greenwood et al. (2016), the performance of 43 schizophrenic patients during shopping in a virtual supermarket was directly compared to the performance during shopping in a real supermarket. The number of correct products, the duration of the shopping trip and the number of aisles reached by the participant were measured during the real and the virtual shopping trip. All three measures were significantly correlated between real live shopping trips and virtual shopping trips. Furthermore, VR measures were significant predictors for real life measures. These studies show exemplarily that behavioral monitoring in VEs seems to be correlated with real life behavior or can at least provide a significant improvement of the prediction accuracy of real life behavior. Despite these promising results, it has to be emphasized that these studies do not show that behavior in VEs necessarily reflect the behavior in daily life (Greenwood et al., 2016). Thus, it is import that future studies test the predictive validity of SOC's behavior in VEs for their behavior in real life. On the other hand, SOCs mostly stated in the P-VRS that they are convinced of the need of avoidance behavior in comparable (real) situations in order to avoid re-offenses. Thus, it can be argued that they experienced the virtual situations as risk situations and not as a “virtual situation” in which one can play around. This may underline the diagnostic and prognostic utility of virtual risk situations.

### 4.5. Limitations

Some limitations have to be mentioned. First, the small sample size does not allow for generalization of the results. In order to provide robust statistical analyses, a much greater sample size is necessary. In addition, an adequate control group, e.g., forensic inpatients with the permission for unsupervised privileges, would enable to proof the validity of the reported results. Therefore, the reported results have to be seen as preliminary.

Another critical point may be that the participants were not able to react freely in the risk situations. They were only able to choose between predefined answers, which may not correspond to the behavior, the individual participant would show under free, real-life situations. Furthermore, the predefined choices were dichotomized with regards to approach and avoidance behavior. Despite the greater workload of providing the participant with more behavior options (e.g., integration of an artificial intelligence for the virtual characters), further studies should enable the participant to behave freely in order to provide a more valid behavioral monitoring. At least, some more behavioral possibilities for SOCs should be integrated in future applications. For example, the possibility to help the children without violating the avoidance strategy (e.g., by asking someone else in the supermarket for help) may provide for the individual SOC more realistic behavioral options. By providing more behavioral alternatives, which are in line with treatment goals would also enhance the benefit of the application for behavioral training during treatment.

It must also be mentioned, that the risk scenarios contain moral dilemmas: the SOC had to decide if he should help the child or if he should avoid helping the child in order to cope with the risk situation. It can be discussed if it is ethically correct to teach SOCs not to help children. The situations contained no possible perilous or life-threatening events for the children, thus avoiding helping the child would be—from our point of view - reasonable from the RP approach point of view. However, it is unclear, how detailed the case of comparable situations were discussed during the therapy from an ethically point of view. Therefore, the behavior of the SOCs may possibly reflect more the result of thinking about this moral dilemma than thinking about the correct coping behavior in risk situations. In future studies, the risk situations should therefore also contain situations, without any moral dilemma for the patient (nevertheless that the current simulated scenarios are reflecting the reality).

At last, current treatment approaches as well as the RP approach go far beyond simply teaching avoidant coping in concrete situations. Risk situations have—from a broader perspective—to be seen as the coincidence of several external as well as internal factors, for example the ability to cope with negative life events or an increasing sexual drive (Laws et al., 2000; Yates et al., 2010). Therefore, coping strategies necessary for successfully coping with situations as shown in the virtual risk situations are not far-reaching enough within the rationale of current treatment approaches. However, it is important to mention, that the risk situations in the current study were explicitly designed for the decision about first unsupervised privileges and therefore do not raise the claim to evaluate the behavior of SOCs in more complex risk situations.

## Author contributions

PF has been involved in the development of the design, programming of the VE, data collection, data analysis, and the preparation of the manuscript. SM has made important contributions to the data collection, data analysis, and the intensive revision of the manuscript. KJ has made significant contributions to development of the design and was involved in drafting and revision of the manuscript. JM supervised and supported the development of the idea and the design and was involved in the drafting and revision of the manuscript. All the authors have read and approved the final manuscript.

### Conflict of interest statement

The authors declare that the research was conducted in the absence of any commercial or financial relationships that could be construed as a potential conflict of interest.
