# Supplementary material for: Behavioral Monitoring of Sexual Offenders Against Children in Virtual Risk Situations: A Feasibility Study
Source: Front Psychol. 2018 Mar 6;9:224. doi: 10.3389/fpsyg.2018.00224 (PMC5845629; doi:10.3389/fpsyg.2018.00224)
Supplement: Supplementary file 1 [file DataSheet1.pdf]

Supplementary Material:
Behavioral monitoring of sexual offenders against children in virtual risk situations: a feasibility study.

Peter Fromberger *, **Sabrina Meyer, Kirsten Jordan and Jürgen L. Müller**
*Correspondence:
Peter Fromberger
peter.fromberger@medizin.uni-goettingen.de

1 S1 INITIAL RATING

1.1 Virtual Environment

The virtual environment for the character rating portrayed an oversimplified greenhouse within a friendly landscape at a sunny day (see Figure S1). Additionally, a convenient gentle background sound (e.g. singing birds) was implemented. The green house had a virtual size of 8.2 x 4.6 meters (about twice the size of the real lab). At one side of the green house, a virtual flat-screen was implemented which presented the instructions and feedback to the participant. Under the virtual flat-screen a simple console was mounted with six number buttons (zero - 6) and an enter button. With the help of this console, the participant was able to press the buttons with a virtual hand in order to rate the virtual characters. The virtual hand was linked to the position data of the Wand, which the participant held in his dominant hand. Thereby, the virtual hand follows all movements of the dominant hand of the subject. The participant could walk freely within the virtual green house. Since the lab area was only one-half of the green house area, each position data point was multiplied by 1.8 (only x- and y-axes).

1.2 Measures

The participants were asked to rate each virtual character on a 6-point Likert-scale with regards to their realism (0 = not realistic at all, 5 = very realistic). The participants were further asked to rate each virtual character on a 6-point Likert-scale with regards to their sexual attractiveness (zero = not sexually attractive, 5 = very sexually attractive). Without the knowledge of the subject, the time from stimulus onset until the end of the sexual attractiveness rating was measured (Viewing Time, VT). The stimulus onset was defined as the first time point, the virtual character was in the field of view of the subject. The VT is a well-established method to assess (deviant) sexual interests (Schmidt et al., 2017). The so-called viewing time effect (VT effect), which is typically found when viewing time is assessed, shows that subjects take a significantly longer time to look at sexually attractive stimuli than at sexually non-attractive stimuli. It was shown, that these effects can be seen with regards to sexual orientation as well as sexual age preference (Harris et al., 1996; Imhoff et al., 2010, 2012; Schmidt et al., 2017). Fromberger et al. (2015) recently demonstrated that the VT effect can also be replicated in high-immersive virtual environments.

1.3 Procedure

When arriving at the lab, a short questionnaire was applied in order to assess basic data (educational level, age, current neurological or psychiatric disorders). The participants were then asked to fill in the Kinsey Scale (Kinsey et al., 1948). Next, the VR equipment and its usage was explained, before the participant was asked to fill in the pre-test of the (Kennedy et al., 1993, SSQ). Afterwards, the participant was equipped with the HMD, the Wand and a headset in the experiment room. The headset allowed the participant to talk with the investigator, who was able to monitor the progress of the experiment in a separate room and to give instructions via the headset. In order to secure, that the cable of the HMD did not trouble the participant, a second investigator was in the experiment room.
After the start of the virtual environment, the participant had the opportunity to become familiar with the virtual environment and the controlling of the environment. The participant was instructed via the headset, that he can freely move within the virtual green house and that he can press the buttons of the console with his virtual hand. He was further told, that he has the task to rate the virtual characters regarding their sexual attractiveness and regarding their realism on a 6-point Likert scale (zero = not sexually attractive at all / not realistic at all, five = very sexually attractive / very realistic). The instruction emphasized that the subjective feeling of the participant was of interest, rather than how he thinks others would feel about the character. Furthermore, he was told that he could look at the virtual characters in more detail, if he walks near to or walk around the virtual character. After all instructions, the participant walked through four test trials (two clothed virtual female and two clothed virtual male characters) in order to get comfortable with the task and the controlling of the experiment. The test trials followed the same rationale as the main trials afterwards. Overall, five trials with stimuli from each category (virtual women, men, boys, and girls) were presented in a fully randomized order. Each trial started with the instructional text on the virtual screen, which prompted the participant to turn around and look at the virtual character behind him. The participant had to look at least one time at the virtual character before he can start rating the character. The virtual character was positioned four meters behind the participant and animated with a neutral idle animation. In order to rate the virtual character, the participant had to go back to the virtual screen and was prompted to touch the number on the console with his virtual hand, which corresponds to his subjective experienced sexual attractiveness. In order to confirm his choice, he additionally had to press a virtual enter button. Without the knowledge of the participant, the time from start of the trial until the end of the attractiveness rating was assessed in each trial (Viewing Time). After the sexual attractiveness rating, the participant was prompted by the screen to rate the realism of the virtual character the same way. A randomized inter-stimulus interval between 10-15 seconds was applied. Every fifth trial, the participant has the opportunity to pause the experiment and to leave the virtual environment. After finishing the experiment, the participant was asked to fill in the post-test of the SSQ, the IPQ, the SPQ and the VRSRS.
Before entering the main experiment phase, the results of the initial rating were automatically analyzed at the individual level in order to identify the virtual adult character with the shortest viewing time and lowest attractiveness rating (most unattractive virtual adult character) and the virtual child character with the longest viewing time and the highest attractiveness rating (most attractive virtual child character).

1.4 Data analysis

Before entering the main experiment phase, the results of the initial rating were automatically analyzed at the individual level in order to identify the virtual adult character with the shortest viewing time and lowest attractiveness rating (most unattractive virtual adult character) and the virtual child character with the longest viewing time and the highest attractiveness rating (most attractive virtual child character). The individual most unattractive adult character was presented during the baseline scenario; the individual most

attractive child was presented during all risk scenarios. In order to validate the identification algorithm, ANOVAS with the within-group factor Condition (Virtual characters presented in the baseline condition vs. virtual characters presented in the scenarios) and the between-group factor Group (NCAs vs. CAs) were performed for all virtual characters chosen for the main experiment with regards to the Viewing Time, the attractiveness rating, and the realism rating.

1.5 Results

Figure S2 shows the means and SDs for the sexual attractiveness rating as a function of condition and subject group. The ANOVA for the attractiveness rating revealed no significant main effect Condition (F(1,11) = .53, p = .480, η^2 = .05), main effect Group (F(1,11) = .37, p = .556, η^2 = .03), or the Group x Condition interaction (F(1,11) = .53, p = .480, η^2 = .05).
Figure **??** shows the means and SDs for the realism rating as a function of condition and subject group. The ANOVA for the realism rating revealed a significant main effect Group (F(1,11) = 5.28, p = .042, η^2 = .32). NOCs (M = 3.57, SD = 1.02) rated all in the baseline and scenarios used virtual characters as significantly more realistic than SOCs (M = 1.92, SD = 1.62). The main effect Condition (F(1,11) = 2.46, p = .145, η^2 = .18) and the interaction Group x Condition (F(1,11) = .18, p = .677, η^2 = .02) revealed no significance.
Figure **??** shows the means and SDs for the viewing time as a function of condition and subject group. The ANOVA for Viewing Time revealed a significant main effect Condition (F(1,11) = 6.37, p = .028, η^2 = .37) and a significant Group x Condition interaction (F(1, 11) = 5.79, p = .035, η^2 = .34). The main effect Group was not significant (F(1, 11) = .04, p = .85, η^2 ¡ .01). Post-hoc t-tests revealed, that SOCs tend to look longer on child characters (M = 31088.82 ms, SD = 14408.53 ms) than on adult characters (M = 17880.66 ms, SD = 10777.90 ms; t(5) = 2.50, p = .055). NOCs showed no difference between the Viewing Time for child characters (M = 23402.16, SD = 12340.17) and adult characters (M = 23090.18 ms, SD = 12155.80 ms; t(6) = 0.15, p = .886).

1.6 Discussion

Main goal of the initial rating was to detect the virtual child character with the highest sexual salience for each individual CA. The developed virtual situation becomes only risky for SOCs, if the presented virtual child character has a high sexual salience and can therefore serve as a trigger. The sexual attractiveness rating of the virtual characters showed no significant difference between the subject groups as well as between adult and child characters. Furthermore, the sexual attractiveness ratings were very low (all ratings were on average below 1 on a 6-point Likert scale between 0 and 5). For healthy homo- and heterosexual subjects, Fromberger et al. (2015) showed that the sexual attractiveness rating of naked virtual adult characters corresponds with the sexual orientation of the subjects. In addition, sexual attractiveness was significantly higher in high-immersive presentation modes (HMD) than in 2D presentation mode (desktop monitor). Possibly, the higher sexual attractiveness ratings in the Fromberger et al. (2015) study in comparison to the current study are a consequence of the more prominent secondary sexual characteristics in the naked virtual characters compared to the clothed characters in the current study. Furthermore, the characters in the current study were animated with neutral poses and facial expressions. Dennis et al. (2014) demonstrated, that heterosexual subjects responded with significant sexual arousal (measured via penis plethysmography) only when virtual adult female characters were depicted as sexually open (e.g. joyful or seductive), rather than sexually closed or neutral. Thus, neutral animations of the virtual characters may have reduced the sexual salience of the stimuli.
The detection of the most sexual salient child character was based on the VT paradigm, which assumes, that the virtual character with the longest VT has also the highest sexual salience (Schmidt et al., 2017).

Recently, it was shown that the viewing time effect can be replicated for heterosexual and homosexual healthy subjects in high-immersive environments and with virtual characters (Fromberger et al., 2015). In the current study, the viewing time paradigm was for the first time used in a high-immersive environment with forensic inpatients and in order to detect the most attractive virtual character at an individual level. To the best of our knowledge, the VT paradigm was until now only used to detect the sexual interest of SOCs based on the average VT revealed by building the mean of the VT for several pictures of the same category (e. g. children vs. adults) (Schmidt et al., 2017). Thus, the validity of the approach used in the study to detect the most sexual salient child character can be criticized. Nevertheless, the results demonstrated that the VT for the child characters was significantly longer than the VT for adult characters used in the risk scenarios in the group of SOCs. NOCs rated the adult and child virtual characters used in the risk scenarios as more realistic than SOCs. However, there was in both groups no significant difference with regards to the subjective realism between adult and child characters. Thus, one can assume that a different realism level of child and adult characters was not the reason for the significant viewing time effect within the SOC group. Therefore, it is reasonable to assume, that the virtual child character used in the risk situations, had the the highest individual sexual salience for each SOC.

REFERENCES

Dennis, E., Rouleau, J.-l., Renaud, P., Kevin, N., and Saumur, C. (2014). A pilot development of virtual stimuli depicting affective dispositions for penile plethysmography assessment of sex offenders. *The Canadian Journal of Human Sexuality* 23, 200–208. doi:10.3138/cjhs.2529

Fromberger, P., Meyer, S., Kempf, C., Jordan, K., and Müller, J. (2015). Virtual viewing time: The relationship between presence and sexual interest in androphilic and gynephilic men. *PLoS ONE* 10. doi:10.1371/journal.pone.0127156

Harris, G. T., Rice, M. E., Quinsey, V. L., and Chaplin, T. C. (1996). Viewing time as a measure of sexual interest among child molesters and normal heterosexual men. *Behaviour Research and Therapy* 34, 389–394. doi:10.1016/0005-7967(95)00070-4

Imhoff, R., Schmidt, A. F., Nordsiek, U., Luzar, C., Young, A. W., and Banse, R. (2010). Viewing time effects revisited: Prolonged response latencies for sexually attractive targets under restricted task conditions. *Archives of Sexual Behavior* 39, 1275–1288. doi:10.1007/s10508-009-9595-2

Imhoff, R., Schmidt, A. F., Weiß, S., Young, A. W., and Banse, R. (2012). Vicarious viewing time: Prolonged response latencies for sexually attractive targets as a function of task- or stimulus-specific processing. *Archives of Sexual Behavior* 41, 1389–1401. doi:10.1007/s10508-011-9879-1

Kennedy, R. S., Lane, N. E., Berbaum, K. S., and Lilienthal, M. G. (1993). Simulator Sickness Questionnaire: An Enhanced Method for Quantifying Simulator Sickness. *The International Journal of Aviation Psychology* 3, 203–220. doi:10.1207/s15327108ijap0303_3

Kinsey, A. C., Pomeroy, W. B., and Martin, C. E. (1948). *Sexual behavior in the human male* (Philadelphia: W.B. Saunders)

Schmidt, A. F., Babchishin, K. M., and Lehmann, R. J. (2017). A Meta-Analysis of Viewing Time Measures of Sexual Interest in Children. *Archives of Sexual Behavior* 46, 287–300. doi:10.1007/s10508-016-0806-3

FIGURES

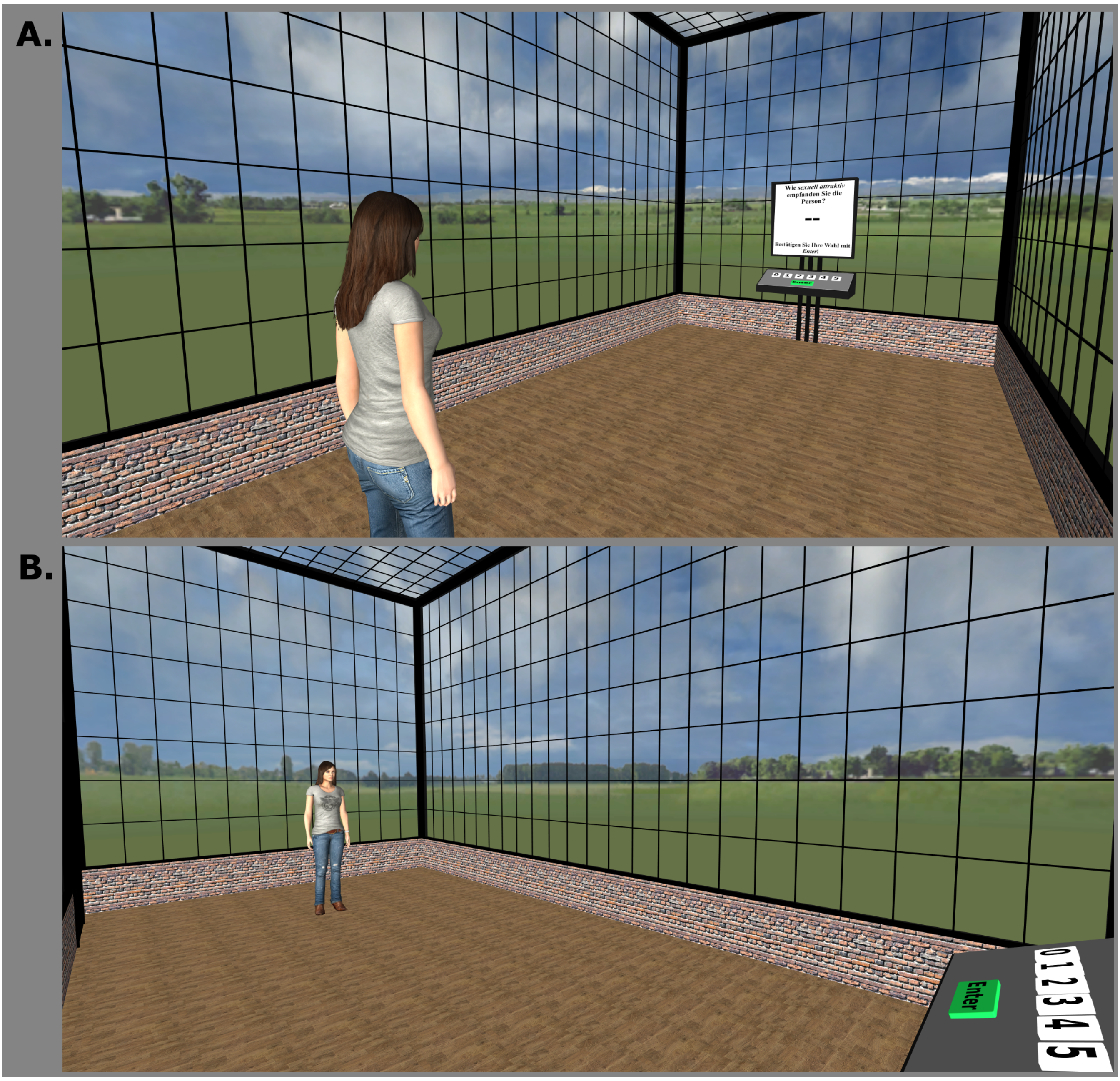

Figure S1: Virtual Environment for the initial rating. (A) View from behind the virtual character. (B) View from the rating console.

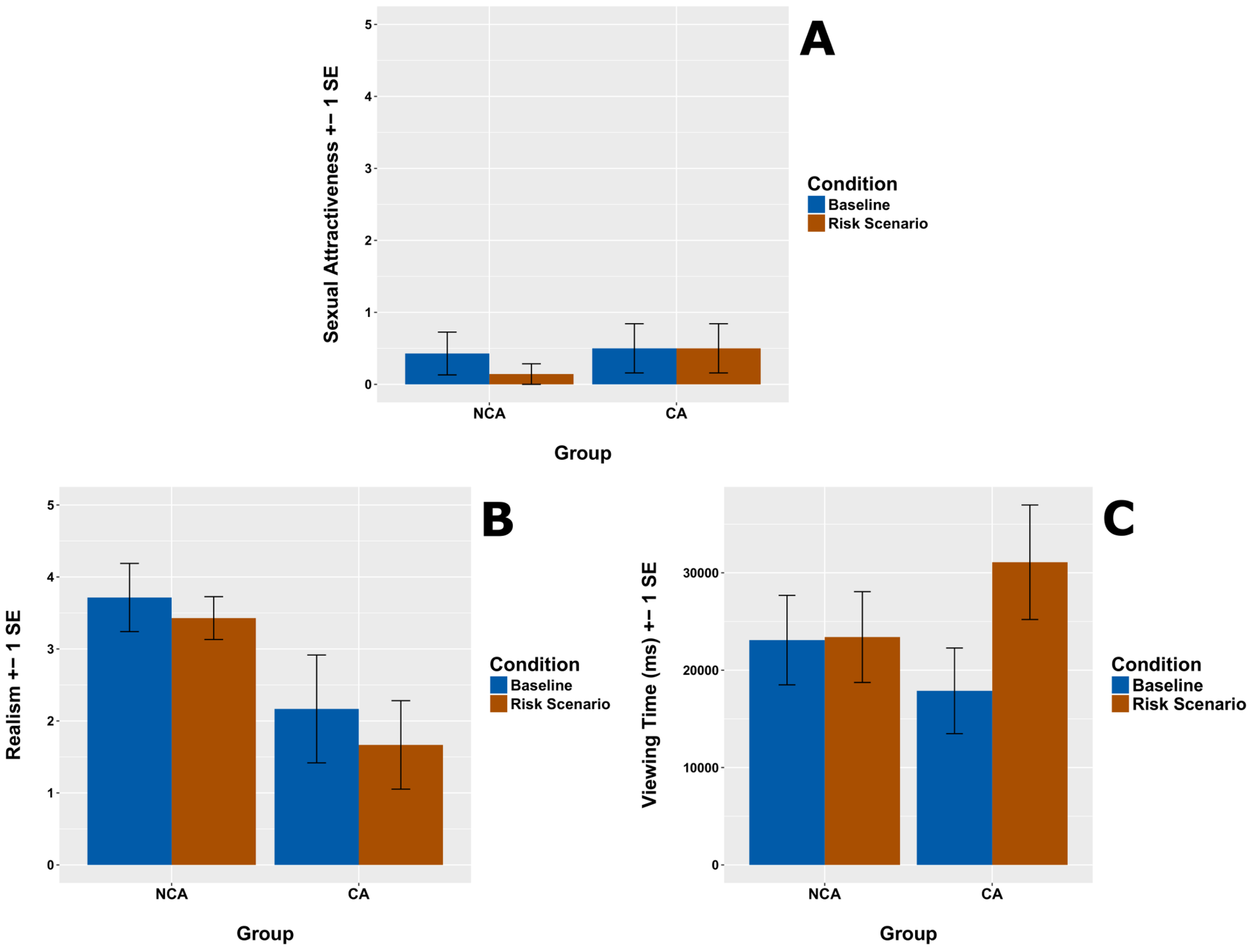

Figure S2: Means and SDs of the sexual attractiveness rating (A), the realism rating (B) and the viewing time (C). The sexual attractiveness rating and the realism rating was based on a 5-point Likert scale. Shown are the values for all virtual characters (adult characters in the baseline condition and child characters in the risk scenarios), used in the main experiment.
