# Supplementary material for: Behavioral Monitoring of Sexual Offenders Against Children in Virtual Risk Situations: A Feasibility Study
Source: Front Psychol. 2018 Mar 6;9:224. doi: 10.3389/fpsyg.2018.00224 (PMC5845629; doi:10.3389/fpsyg.2018.00224)
Supplement: Supplementary file 2 [file DataSheet2.pdf]

Supplementary Material:
Behavioral monitoring of sexual offenders against children in virtual risk situations: a feasibility study.

Peter Fromberger *, Sabrina Meyer, Kirsten Jordan and Jürgen L. Müller

*Correspondence:
Peter Fromberger
peter.fromberger@medizin.uni-goettingen.de

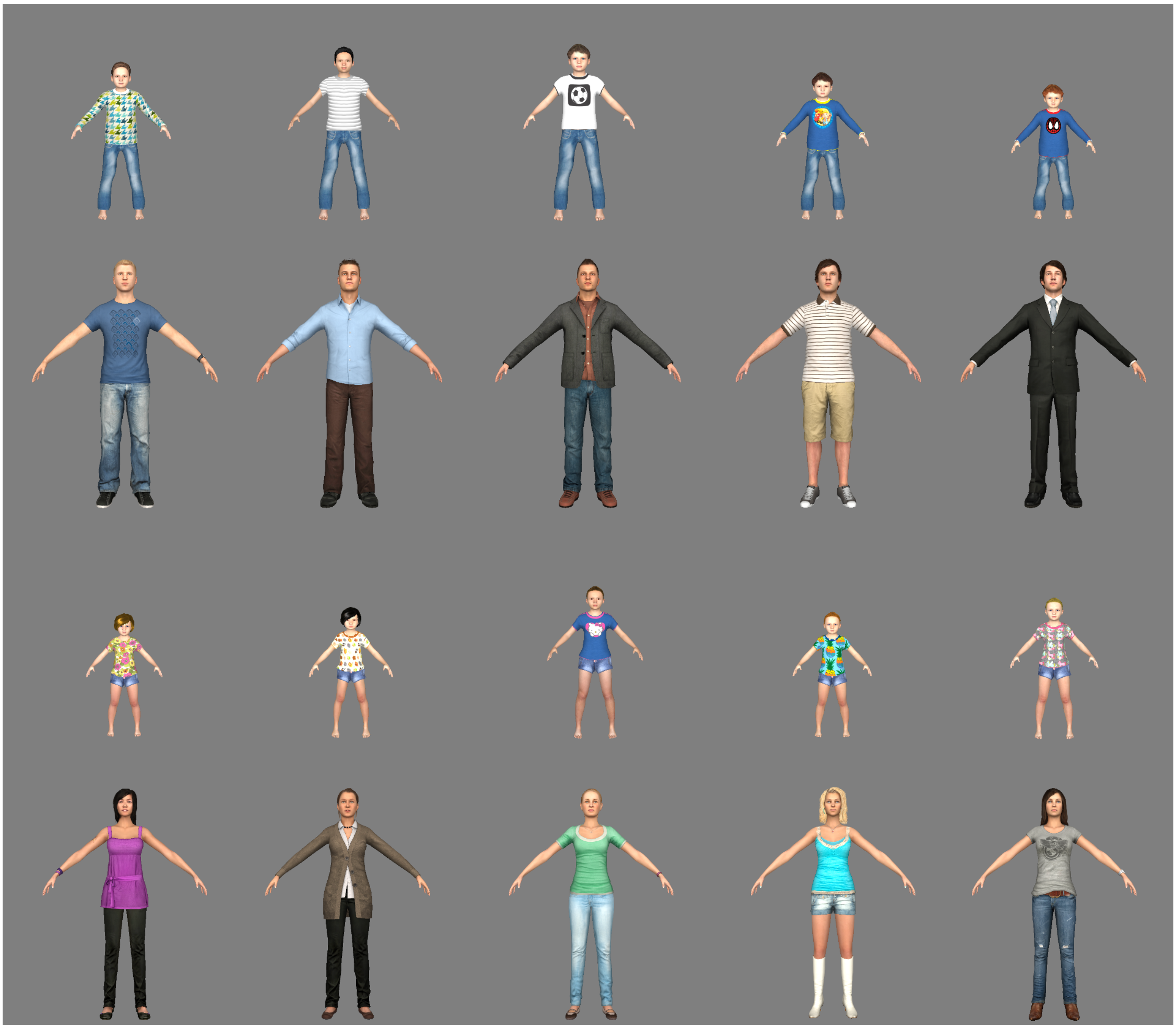

Figure S3: Overview of the virtual characters. Adult characters are from the Complete Characters HD Set (Rocketbox Studios GmbH, Hannover, Germany). Child characters were modeled by the Clinic of Psychiatry and Psychotherapy - Forensic Psychiatry, Human Medical Center Göttingen, Georg-August-University of Göttingen. All characters are fully rigged and animated.
