# Supplementary material for: Behavioral Monitoring of Sexual Offenders Against Children in Virtual Risk Situations: A Feasibility Study
Source: Front Psychol. 2018 Mar 6;9:224. doi: 10.3389/fpsyg.2018.00224 (PMC5845629; doi:10.3389/fpsyg.2018.00224)
Supplement: Supplementary file 3 [file DataSheet3.pdf]

Supplementary Material: Behavioral monitoring of sexual offenders against children in virtual risk situations: a feasibility study.

Peter Fromberger *, **Sabrina Meyer, Kirsten Jordan and Jürgen L. Müller**

*Correspondence:
Peter Fromberger
peter.fromberger@medizin.uni-goettingen.de

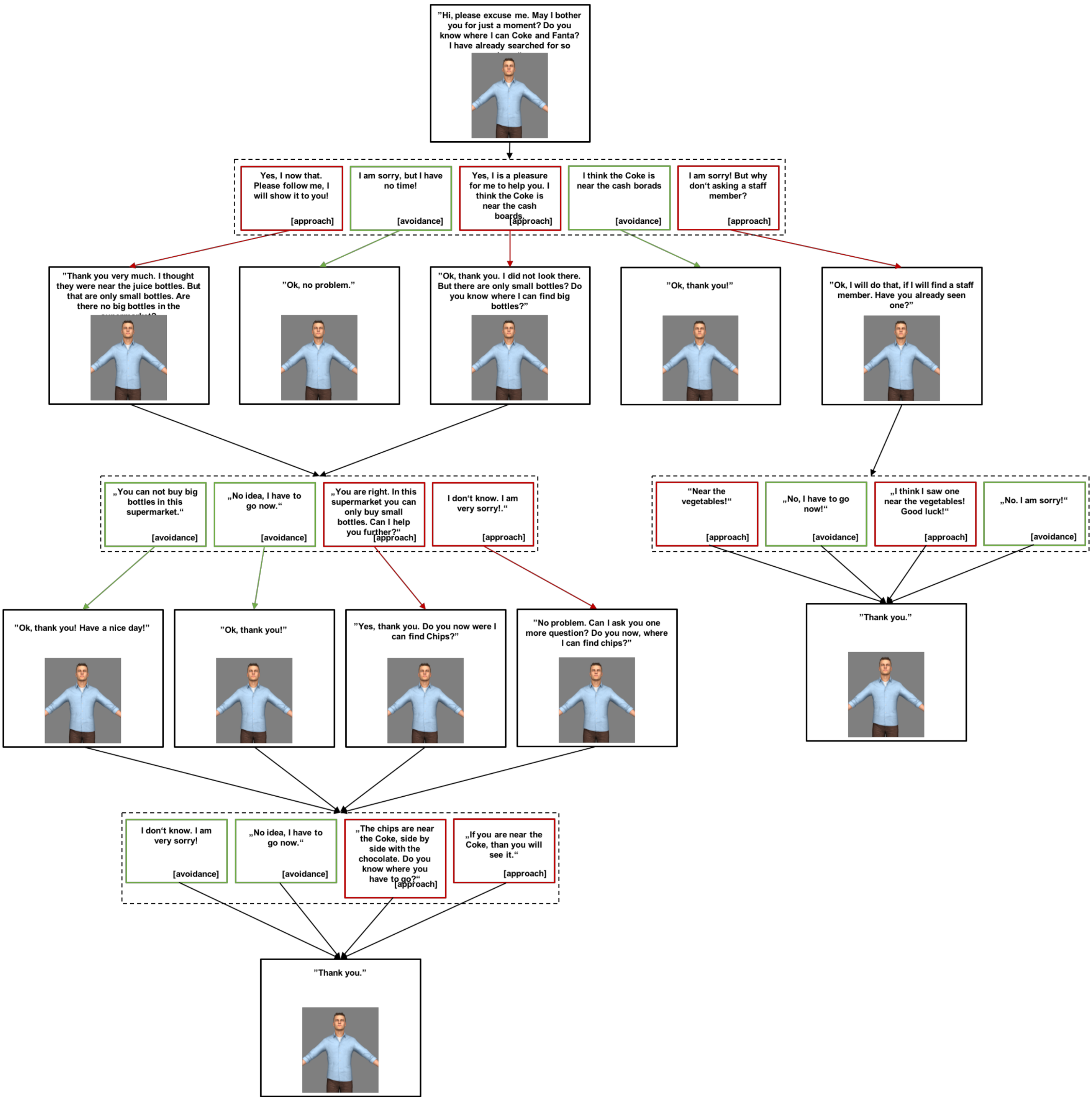

Figure S4: Interaction tree of the baseline scenario. In the baseline scenario, the sexually most unattractive virtual male character forced the subject to interact. The interaction with the virtual adult character was not avoidable. Approach behavior is marked with red squares, avoidance behavior with green squares.
