# Supplementary material for: Behavioral Monitoring of Sexual Offenders Against Children in Virtual Risk Situations: A Feasibility Study
Source: Front Psychol. 2018 Mar 6;9:224. doi: 10.3389/fpsyg.2018.00224 (PMC5845629; doi:10.3389/fpsyg.2018.00224)
Supplement: Supplementary file 4 [file DataSheet4.pdf]

Supplementary Material:
Behavioral monitoring of sexual offenders against children in virtual risk situations: a feasibility study.

Peter Fromberger *, **Sabrina Meyer, Kirsten Jordan and Jürgen L. Müller**

*Correspondence:
Peter Fromberger
peter.fromberger@medizin.uni-goettingen.de

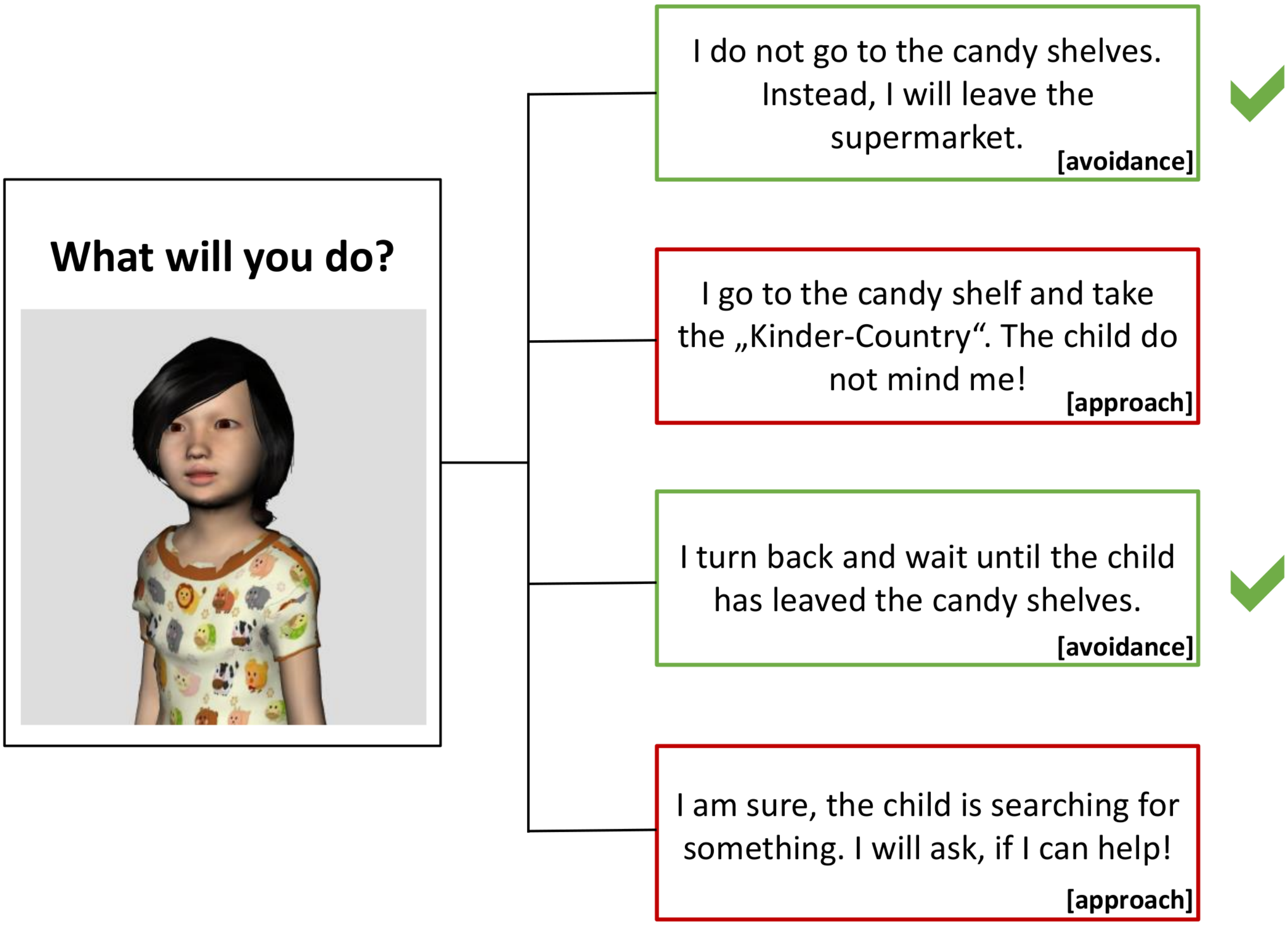

Figure S5: Interaction tree of risk scenario one. In virtual risk scenario one, a virtual child character walks to the candy shelves. The subject has to choose one of four predefined behaviors. Approach behavior is marked with red squares, avoidance behavior with green squares. Green check marks represent behavior in line with the traditional Relapse-Prevention approach.
