# Supplementary material for: Behavioral Monitoring of Sexual Offenders Against Children in Virtual Risk Situations: A Feasibility Study
Source: Front Psychol. 2018 Mar 6;9:224. doi: 10.3389/fpsyg.2018.00224 (PMC5845629; doi:10.3389/fpsyg.2018.00224)
Supplement: Supplementary file 6 [file DataSheet6.pdf]

Supplementary Material: Behavioral monitoring of sexual offenders against children in virtual risk situations: a feasibility study.

Peter Fromberger*, **Sabrina Meyer, Kirsten Jordan and Jürgen L. Müller**

*Correspondence:
Peter Fromberger
peter.fromberger@medizin.uni-goettingen.de

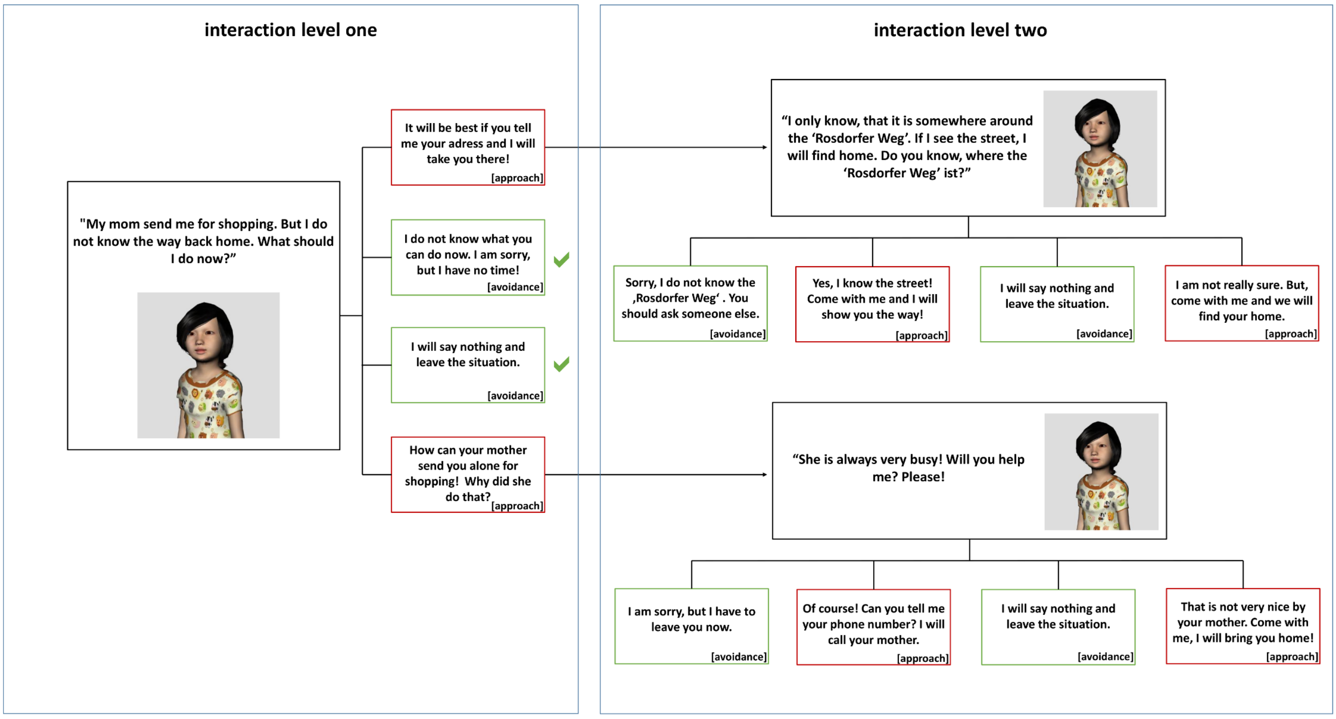

Figure S7: Interaction tree of risk scenario three. In virtual risk scenario three, a virtual child character asked the subject for help. The interaction with the virtual child character was not avoidable. Two interaction levels are possible, if the subject chose approach behavior at the first level. Approach behavior is marked with red squares, avoidance behavior with green squares. Green check marks represent behavior in line with the traditional Relapse-Prevention approach.
